# Bioguided Identification of Polymethoxyflavones as Novel Vascular Ca_V_1.2 Channel Blockers from Citrus Peel

**DOI:** 10.3390/molecules29235693

**Published:** 2024-12-02

**Authors:** Anna Ramunno, Rosa Maria Vitale, Pietro Amodeo, Carlo Crescenzi, Alice Panti, Paolo Fiorenzani, Michele De Luca, Umile Gianfranco Spizzirri, Donatella Restuccia, Francesca Aiello, Fabio Fusi

**Affiliations:** 1 Department of Pharmacy, University of Salerno, 84084 Fisciano, SA, Italy; aramunno@unisa.it (A.R.); carlo.crescenzi@unisa.it (C.C.); 2 Institute of Biomolecular Chemistry-National Research Council (ICB-CNR), 80078 Pozzuoli, NA, Italy; rmvitale@icb.cnr.it (R.M.V.); pamodeo@icb.cnr.it (P.A.); 3 Department of Life Sciences, University of Siena, 53100 Siena, TS, Italy; alice.panti1@unisi.it; 4 Department of Biotechnology, Chemistry, and Pharmacy, University of Siena, 53100 Siena, TS, Italyfabio.fusi@unisi.it (F.F.); 5 Department of Pharmacy, Health and Nutritional Sciences, University of Calabria, 87036 Arcavacata di Rende, CS, Italy; michele.deluca@unical.it; 6 Ionian Department of Law, Economics and Environment, University of Bari Aldo Moro, 74123 Taranto, TA, Italy; umile_gianfranco.spizzirri@unical.it; 7 Department of Management, University of Roma La Sapienza, 00161 Rome, RM, Italy; donatella.restuccia@uniroma1.it

**Keywords:** polymethoxyflavones, Ca_V_1.2 channel blockers, docking studies, multivariate curve resolution

## Abstract

The huge amount of citrus peel produced worldwide represents an economic burden for society. However, this agricultural by-product is a rich source of natural molecules, potentially endowed with interesting pharmacological activities. In this regard, we decided to investigate if the polymethoxyflavones contained in citrus peel waste could be exploited as novel vasorelaxant agents. A hydroalcoholic blond orange (*Citrus sinensis*) peel extract, obtained by ultrasonication, was partitioned in dichloromethane. Column chromatography allowed for the isolation of four polymethoxyflavones, namely, scutellarein tetramethyl ether, nobiletin, tangeretin, and sinensetin, identified by nuclear magnetic resonance (NMR) spectroscopy and UPLC-HRMS/MS and confirmed by multivariate curve resolution of NMR fractional spectra. The four molecules showed interesting in vitro vasorelaxant activity, at least, in part, due to the blockade of smooth muscle Ca_V_1.2 channels. Molecular modeling and docking analysis elucidated the binding mode of the polymethoxyflavones at the homology model of the rat Ca_V_1.2c subunit and provided the structural basis to rationalise the highest activity of scutellarein tetramethyl ether in the set and the dramatic effect of the additional methoxy group occurring in nobiletin and sinensetin. In conclusion, citrus peel can be considered a freely available, valuable source of vasoactive compounds worthy of pharmaceutical and/or nutraceutical exploitation.

## 1. Introduction

Polymethoxyflavones (PMFs) are structurally characterized by a benzo-γ-pyrone (15-C) skeleton, consisting of two benzene rings (6-C) linked by a linear three-carbon chain (3-C) with a carbonyl group at the C4-position, presenting several methoxy substituents [1]. The 60 types of PMFs identified so far are classified as dimethoxy, trimethoxy, tetramethoxy, pentamethoxy, hexamethoxy, and heptamethoxyflavones, as well as several hydroxy polymethoxylate ones (OH-PMFs) [2]. PMFs are more lipophilic than OH-PMFs, allowing them to cross the phospholipid membrane [3]. Their metabolism is influenced by the number and position of methoxy groups. However, the knowledge of the metabolic fate of different PMF members is still unsatisfactory, and additional in-depth ADME studies of this class of compounds are needed.

A plethora of in vivo and in vitro investigations demonstrate that PMFs possess several biological properties, including cytotoxicity, anti-inflammatory activity, neuroprotection, regulation of metabolic syndrome, skin protection, and interesting antioxidant effects [3,4,5,6,7]. For example, nobiletin and 5-demethylnobiletin have been recently proposed as valuable protective agents against diseases affecting not only the central nervous system but also the peripheral one [8,9] and against cancer because they show various beneficial effects while protecting normal tissues [10]. The number and position of methoxy groups impact the pharmacological profile of this class of compounds. For example, a structure–activity relationship (SAR) analysis performed to define the requirements underpinning the antioxidant activity of PMFs, demonstrated that 5-demethylnobiletin and 5-demethyltangeretin are more potent than their parent compounds nobiletin and tangeretin, respectively [11]. PMFs are found almost exclusively in the genus *Citrus* (Rutacee), particularly in the peels, and have been used in traditional medicine for thousands of years to treat several diseases [12]. Considering the remarkable amount of peels generated by the industrial transformation of citrus fruits, the PMFs contained in this waste represent a sustainable source of bioactive compounds that profit from the circular economy [13].

Currently, systematic reports on the extraction, separation, and pharmacological assessment of citrus PMFs are still scarce. Each method has limitations and advantages concerning efficiency, selectivity, yield, cost, property, and safe usage. For example, the best extraction efficiency can be obtained using a 75% ethanol solution. Furthermore, conventional and innovative methods can significantly affect the composition, structure, and biological activity of the total extract. Ultrasound-assisted extraction (UAE), for example, is a methodology capable of valorizing agricultural by-products, with better extraction efficiency, shorter extraction time, and higher yields than traditional extraction methods [14]. Furthermore, the multivariate curve resolution (MCR) technique allows for accurate metabolomics analysis. In fact, MCR techniques can efficiently analyze complex spectral datasets generated from various analytical methods [15]. Additionally, techniques such as multivariate curve resolution–alternating least squares (MCR-ALS) enable the deconvolution and integration of spectral data without prior information, allowing for the extraction of detailed metabolite information for further investigations [15]. This approach assists in determining metabolite concentrations, patterns, and variations across different samples, thereby enhancing the comprehension of metabolic profiles [16].

Querying PubMed for titles including “citrus peel”, we retrieved more than 150 papers. However, studies investigating the cardiovascular effects of extracts from this agricultural waste are scarce. Since flavonoids have been reported to target Ca_V_1.2 channels and elicit cardiovascular protection [1,17], we pursued a multidisciplinary approach to evaluate if blond orange peels can provide this kind of biologically functional compound, i.e., a series of PMFs. Once extracted, isolated, structurally identified, and assessed in vitro and in silico, these molecules performed as effective vasorelaxant agents capable of blocking Ca_V_1.2 channels, thus representing valuable compounds deserving pharmaceutical and/or nutraceutical exploitation as novel weapons in the cardiovascular arsenal.

## 2. Results

### 2.1. Citrus Extracts Display Vasorelaxant Activity

**Figure 1:**
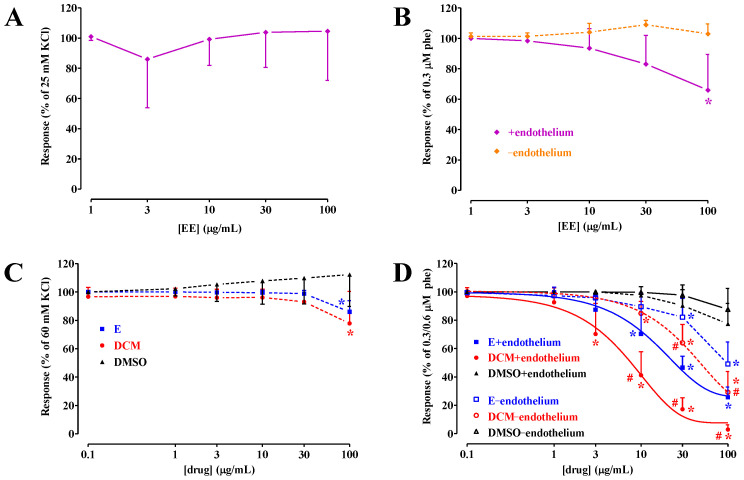
Citrus ethanolic extract, as well as *n*-hexane and dichloromethane phases, are effective against phenylephrine-induced but not KCl-induced contraction. Rings stimulated either (**A**) by 25 mM or (**C**) 60 mM KCl (in the absence of a functional endothelium) or (**B**,**D**) by 0.3–0.6 µM phenylephrine [phe; endothelium-intact (+endothelium) or -deprived (−endothelium)] were challenged with cumulative concentrations of the citrus ethanolic extract (EE), *n*-hexane (E), or dichloromethane (DCM) phases. The effect of the solvent dimethyl sulfoxide (DMSO) is also shown. In the ordinate scale, relaxation is reported as a percentage of the initial tension induced by either KCl or phenylephrine. ((**A**) KCl 860 ± 322 mg, n = 3; (**B**) phe + endo 238 ± 136 mg, n = 4, and phe−endo 667 ± 75 mg, n = 3, *p* = 0.0046; (**C**) KCl DCM 640 ± 392 mg, n = 5, E 740 ± 237 mg, n = 5, and DMSO 648 ± 364 mg, n = 5, *p* = 0.8744; (**D**) phe + endo DCM 738 ± 364 mg, n = 5, E 1186 ± 555 mg, n = 5, and DMSO 760 ± 439 mg, n = 6, *p* = 0.2467; phe − endo DCM 1092 ± 217 mg, n = 5, E 880 ± 718 mg, n = 5, and DMSO 1069 ± 467 mg, n = 8, *p* = 0.7568; KCl, phe + endo, and phe − endo for DMSO *p* = 0.2182; for DCM *p* = 0.1199; for E *p* = 0.4370]. Data points represent the mean ± SD (n = 3–8). (**B**) * *p* < 0.05 vs. −endothelium, (**C**) * *p* < 0.05 vs. DMSO, and (**D**) * *p* < 0.05 vs. DMSO and # *p* < 0.05 vs. E, two-way ANOVA and the Bonferroni multiple comparison test.

A preliminary series of experiments was performed to assess whether the citrus extracts, namely, the ethanolic extract and the subsequent *n*-hexane and dichloromethane phases, contained compounds capable of inducing in vitro vasorelaxation. Rings were either depolarized by moderate concentrations of KCl to disclose any potential K^+^ channel opening activity [18] or stimulated by phenylephrine, an α_1_-selective agonist used to mimic the endogenous vasoconstrictor adrenaline. As shown in Figure 1A,B, the ethanolic extract was ineffective on both contractions; only a modest yet significant vasorelaxation was recorded in rings with an intact endothelium (Figure 1B).

Both dichloromethane and *n*-hexane phases showed similar, modest spasmolytic activity in rings depolarized by high KCl concentrations (Figure 1C and Table 1) to disclose any potential Ca_V_1.2 channel-blocking activity [18]. However, they were significantly more effective in preparations stimulated by phenylephrine. Under these experimental conditions, the dichloromethane phase was significantly more effective than the *n*-hexane one (Figure 1D and Table 1). Interestingly, both phases contained components capable of eliciting endothelium-dependent relaxation as, in the absence of a functional endothelium, their potency and efficacy were significantly lower than those recorded in intact rings (Table 1).

**Figure 2:**
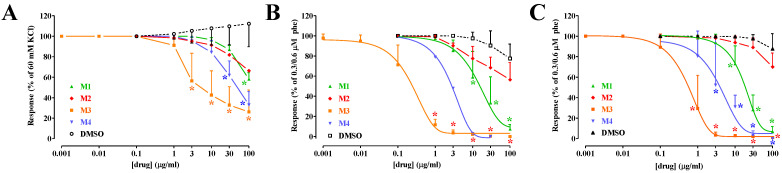
Citrus **M1** to **M4** fractions antagonized KCl- and phenylephrine-induced contraction. Rings stimulated either (**A**) by 60 mM KCl (in the absence of a functional endothelium) or (**B**,**C**) by 0.3/0.6 µM phenylephrine (phe), (**B**) endothelium-intact or (**C**) -deprived, were challenged with cumulative concentrations of the citrus fractions **M1**–**M4**. The effect of solvent (DMSO) is also shown. In the ordinate scale, relaxation is reported as a percentage of the initial tension induced by either KCl or phenylephrine. [(**A**) KCl DMSO 648 ± 364 mg, n = 5, **M1** 602 ± 231 mg, n = 5, **M2** 1630 mg, n = 2, **M3** 1822 ± 436 mg, n = 5, **M4** 507 ± 276 mg, n = 3; (**B**) phe + endo DMSO 760 ± 439 mg, n = 5, **M1** 956 ± 499 mg, n = 5, **M2** 698 ± 383 mg, n = 5, **M3** 1244 ± 418 mg, n = 5, **M4** 815, n = 2; (**C**) phe-endo DMSO 1092 ± 217 mg, n = 8, **M1** 862 ± 335 mg, n = 5, **M2** 1080 ± 636 mg, n = 3, **M3** 1623 ± 278 mg, n = 6, **M4** 977 ± 757, n = 3; *p* = 0.1033; KCl, phe + endo, and phe-endo for DMSO see legend to Figure 1; KCl, phe + endo, and phe-endo for **M1** *p* = 0.3291; for **M3** *p* = 0.0815]. Data points represent the mean ± SD (n = 3–5). * *p* < 0.05 vs. DMSO, two-way ANOVA, and the Bonferroni multiple comparison test. **M2** (**A**) and **M4** (**B**) were excluded from the statistical analysis because they included only two replicates.

The DCM phase was further investigated by carrying out a tentative column chromatography separation, which provided four isolated fractions (M1–M4), assessed in a second series of experiments. As shown in Figure 2, M3 and M4 were the most potent vasorelaxant fractions (Table 1). In general, efficacy and potency were higher against phenylephrine- than against KCl-induced contraction. Surprisingly, the components of the DCM phase endowed with endothelium-dependent vasorelaxant activity (see Figure 1D and Table 1) were lost during column chromatography separation and, therefore, not present in the M1–M4 fractions, which showed comparable potency and efficacy both in the presence or absence of an intact endothelium (Table 1).

### 2.2. Chemical Characterization of M3 and M4

A preliminary investigation by HRMS and ^1^H NMR was performed on both M3 and M4 fractions (Figures S1–S5, SI). The HRMS spectrum of M3 showed three peaks with *m*/*z* 343.1167, 373.1270, and 403.1376, which can be related to a tetramethoxyflavone [C_19_H_19_O_6_]^+^ (mass error:−2.62 ppm), a pentamethoxyflavone [C_20_H_21_O_7_]^+^ (mass error: −3.22 ppm), and an hexamethoxyflavone [C_21_H_23_O_78_]^+^ (mass error: 2.73 ppm), respectively (Figure S1, SI). The ^1^H NMR spectrum of M3 evidenced diagnostic peaks for scutellarein TME, nobiletin, tangeretin, and sinensetin (Figures S2–S4, SI). On the contrary, the ^1^H NMR spectrum of M4 indicated the presence of sinensetin alone (Figure S5, SI).

**Figure 3:**
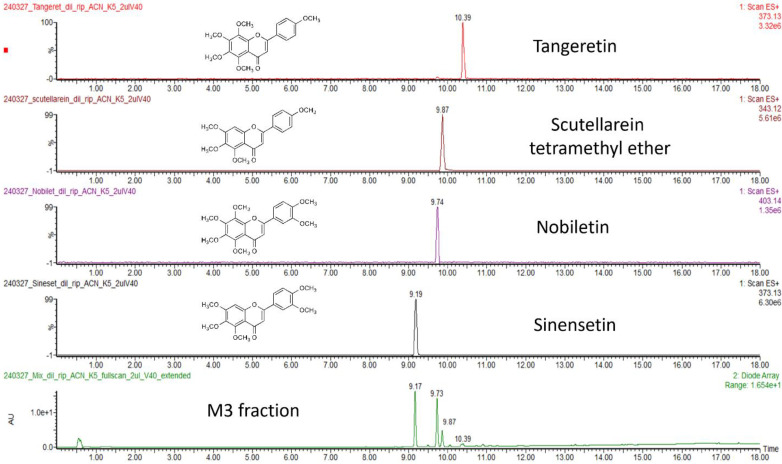
UPLC-PDA-MS chromatograms of **M3** fraction and single PMF standards.

The M3 fraction was unequivocally characterized by UPLC-HRMS/MS. Each component of M3 was separated using reversed-phase under the condition described in the experimental section, and the four major components of the mixture were identified with a hybrid linear ion trap-orbitrap mass analyzer (summarized data and collected spectra are reported in Supporting Information Table S1 and Figure S6). Exact quantitative analysis was performed by injecting the mixture and appropriate standards using a UPLC system (Accela, Thermo Scientific, San Jose, CA, USA) combined with a photodiode array (PDA) detector (Acquity, Waters, Millford, MA, USA) and a mass spectrometry detector (LTQ Orbitrap XL, Thermo Scientific, San Jose, CA, USA) (Figure 3). As a result, nearly 85% was accounted for by the four most abundant PMFs: sinensetin (41%), nobiletin (32%), scutellarein TME (9%), and tangeretin (2%).

To further confirm peak identity and peak purity, in-source fragmentation was obtained by increasing cone voltage to 40 V, and UV spectra were collected in the range of 190–400 nm. The spectra of the single standards perfectly overlapped with those of the M3 fraction components (Figure S7, SI).

### 2.3. Multivariate Identification of Metabolites in Fraction M3 via MCR-ALS Algorithm

**Figure 4:**
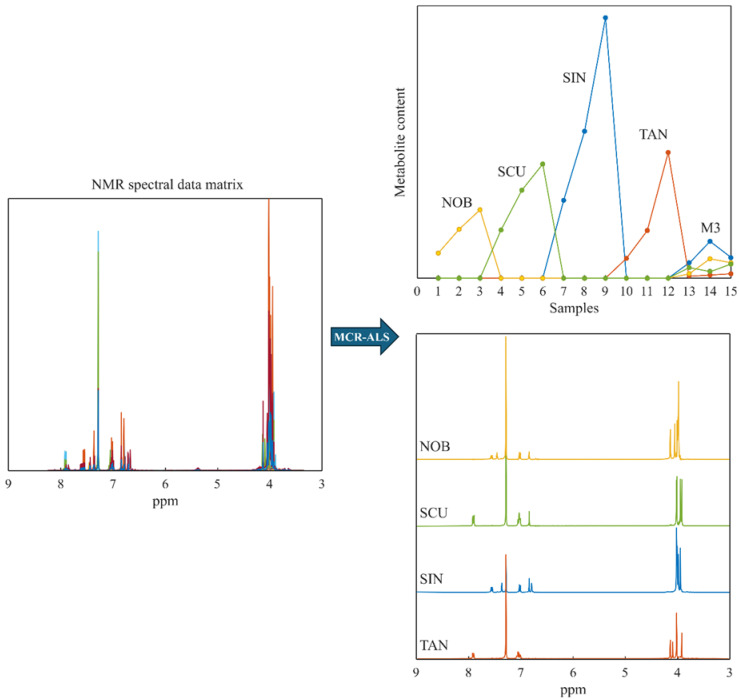
Composition profiles and pure spectra obtained by MCR-ALS analysis on the spectral matrix: nobiletin (NOB), scutellarein TME (SCU), sinensetin (SIN), and tangeretin (TAN).

The multivariate curve resolution (MCR) was applied to extract the peak areas of resolved metabolites from the NMR spectra [19]. After appropriate data pre-treatment, the aligned spectra were resolved using specific constraints, enabling the alternating least squares (ALS) algorithm to identify the chemical species present in the mixture M3 obtained after extraction and fractionation procedures. A spectral matrix was organized by including spectra from the four standards—scutellarein TME, nobiletin, tangeretin, and sinensetin—along with the M3 fraction (all samples in triplicate, 15 samples described by 7029 ppm variables). In the initial step, the number of substances within the mixture was estimated using the singular value decomposition (SVD) method. Principal component analysis confirmed the presence of four chemical species in the mixture [20]. Subsequently, the MCR-ALS algorithm was employed to resolve the M3 mixture and identify the spectral contributions of the four identified components. Throughout the processing cycles, selectivity constraints guided the algorithm, enabling it to distinguish between the spectra of the standards and search for them, if present, within the M3 mixture [20]. The resolution successfully identified the four metabolites in the M3 fraction, accounting for 99% of the spectral variance present in the experimental data. The spectra calculated by the ALS algorithm perfectly matched those of the standards (Figure 4).

### 2.4. The Four Pure PMFs Inhibited Both KCl- and Phenylephrine-Induced Contraction

This series of experiments was performed to investigate the effects of scutellarein TME, tangeretin, nobiletin, and sinensetin on rings stimulated by either high KCl concentrations or the α_1_ adrenergic receptor agonist phenylephrine.

**Figure 5:**
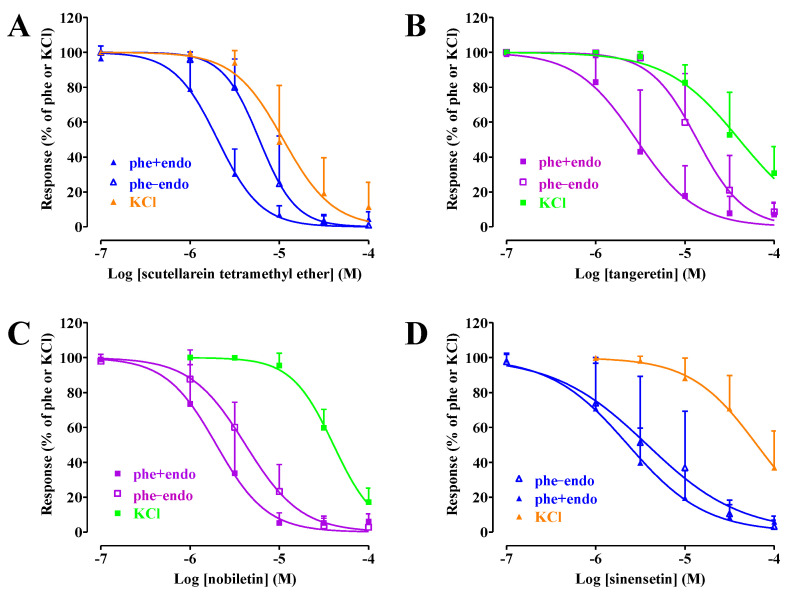
Inhibition of KCl- and phenylephrine-induced contraction by scutellarein TME, tangeretin, nobiletin, and sinensetin. Rings stimulated by either 60 mM KCl (in the absence of a functional endothelium) or by 0.3 µM phenylephrine [phe; endothelium-intact (+endo) or -deprived (−endo)] were challenged with cumulative concentrations of (**A**) scutellarein TME, (**B**) tangeretin, (**C**) nobiletin, and (**D**) sinensetin. In the ordinate scale, relaxation is reported as a percentage of the initial tension induced by KCl or phenylephrine. [(**A**) KCl 1292 ± 460 mg, n = 5, phe + endo 954 ± 456 mg, n = 5, phe − endo 1584 ± 645 mg, n = 5; *p* = 0.2098; (**B**) KCl 1556 ± 448 mg, n = 5, phe + endo 1267 ± 590 mg, n = 6, phe − endo 2138 ± 1145 mg, n = 5; *p* = 0.2105; (**C**) KCl 2178 ± 618 mg, n = 5, phe + endo 1395 ± 504 mg, n = 8, phe − endo 1680 ± 382 mg, n = 5; *p* = 0.0515; (**D**) KCl 2082 ± 560 mg, n = 5, phe + endo 1016 ± 406 mg, n = 5, phe − endo 1898 ± 905 mg, n = 5; *p* = 0.0542]. Data points represent the mean ± SD (n = 4–8).

In rings stimulated by 60 mM KCl, which causes membrane depolarization and Ca_V_1.2 channel opening, all the compounds assessed caused concentration-dependent relaxation with pIC_50_ values (estimated for tangeretin and sinensetin) of 4.91 ± 0.35 (scutellarein TME, n = 5), 4.40 ± 0.28 (tangeretin, n = 5), 4.39 ± 0.11 (nobiletin, n = 5), and 4.23 ± 0.26 (sinensetin, n = 5) (Figure 5).

All the compounds assessed in endothelium-denuded preparations caused concentration-dependent relaxation of the α_1_ adrenergic receptor agonist phenylephrine-induced contraction. Scutellarein TME showed a pIC_50_ value of 5.24 ± 0.20 (n = 5), which was not significantly different from that recorded in depolarized rings (*p* = 0.1145). Conversely, tangeretin (4.82 ± 0.21; n = 5; *p* = 0.0294), nobiletin (5.38 ± 0.19; n = 5; *p* < 0.0001), and sinensetin (5.45 ± 0.58; n = 8; *p* = 0.0011) were more potent vasorelaxant agents on the phenylephrine-induced contraction (Figure 5). The presence of an intact endothelium significantly increased the potency of scutellarein TME (pIC_50_ value of 5.72 ± 0.21, n = 5; *p* = 0.0097 vs. endothelium-deprived rings) and tangeretin (5.26 ± 0.38, n = 6; *p* = 0.0051), partially improved that of nobiletin (5.75 ± 0.37, n = 8; *p* = 0.0628), without affecting that of sinensetin (5.63 ± 0.40, n = 8; *p* = 0.5269) (Figure 5).

### 2.5. The M3 Extract and the Four PMFs Inhibited I_Ba1.2_

**Figure 6:**
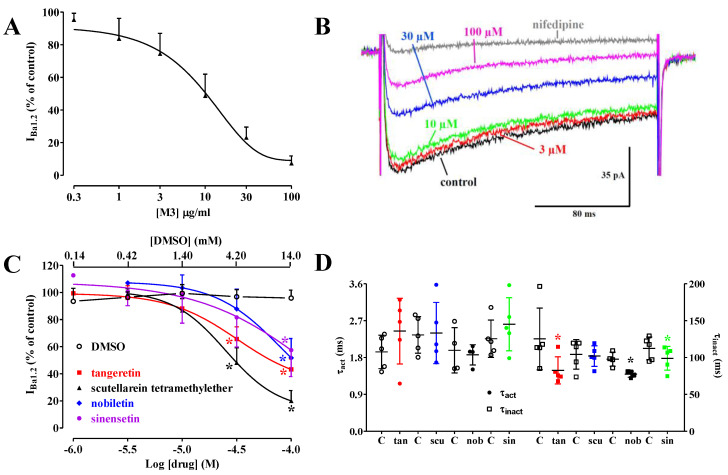
**M3** fraction, scutellarein TME, tangeretin, nobiletin, and sinensetin inhibited I_Ba1.2_ in single tail artery myocytes. Concentration-dependent effect of (**A**) **M3** fraction and (**C**) vehicle (DMSO), scutellarein TME, tangeretin, nobiletin, and sinensetin on I_Ba1.2_. On the ordinate scale, the current amplitude is reported as a percentage of the value recorded just before the addition of the first drug concentration. Data points represent the mean ± SD (n = 4–8). * *p* < 0.05 vs. DMSO, two-way ANOVA, and the Bonferroni multiple comparison test. (**B**) Traces (representative of 5 similar experiments) of I_Ba1.2_, elicited by 250-ms clamp pulses to 10 mV from a V_h_ of −50 mV, measured in the absence (control) or presence of various concentrations of scutellarein TME. The effect of 10 µM nifedipine is also shown. (**D**) Effect of flavonoids on I_Ba1.2_ kinetics of single tail artery myocytes. Time constants for activation (τ_act_) and inactivation (τ_inact_) were measured in the absence or presence of 100 µM tangeretin (tan), scutellarein TME (scu), nobiletin (nob), or sinensetin (sin). Lines and bars represent the mean ± SD (n = 5). * *p* < 0.05 vs. control (**C**), Student’s *t*-test for paired data.

As stated above, vasorelaxation of KCl-induced contraction is a hallmark of Ca_V_1.2 channel blockers. Therefore, a preliminary series of experiments was performed to evaluate the potential Ca^2+^ antagonistic activity of the M3 fraction. Figure 6A shows the effect of cumulative concentrations of the extract on I_Ba1.2_ recorded with depolarizing steps to 10 mV from a V_h_ of −50 mV. M3 inhibited the current amplitude in a concentration-dependent manner with an IC_50_ value of 13.0 ± 4.6 µg/mL (n = 5).

In a second series of experiments, the four PMFs were also assessed individually on I_Ba1.2_. As shown in Figure 6B,C, they inhibited the current in a concentration-dependent manner. Scutellarein TME and tangeretin showed an estimated pIC_50_ value of 4.48 ± 0.13 (n = 5) and 4.18, respectively, whereas sinensetin and nobiletin, at the maximum concentration assessed (100 µM) caused a 50% reduction in current amplitude (Figure 6C).

I_Ba1.2_ evoked at 10 mV from a V_h_ of −50 mV activated and then declined with time courses that could be fitted by a mono-exponential function. None of the pure compounds assessed affected the τ of activation. However, tangeretin, sinensetin, and nobiletin significantly accelerated that of inactivation (Figure 6D).

### 2.6. Homology Modeling of Rat Ca_V_1.2 α_1C_ Subunit and Docking Studies on the Polymethoxyflavones

**Figure 7:**
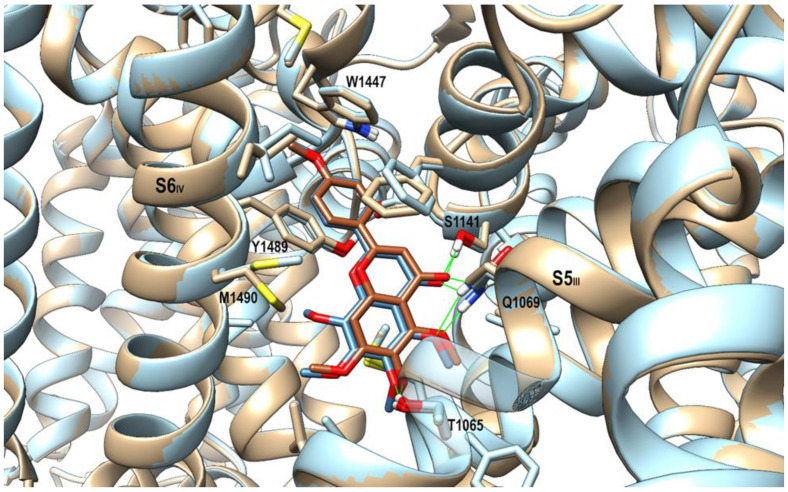
The best fit at the level of protein backbone for the representative docking poses of tangeretin (colored in slate blue) and scutellarein TME (colored in sienna). A stick representation is used for heavy atoms of the ligand and protein side chains within 5 Å of the ligand (colored in light blue and tan for tangeretin and scutellarein TME complexes, respectively. Protein backbone atoms are represented as ribbons colored according to the side chains, using half-transparency in correspondence with the ligand. Hydrogen, nitrogen, oxygen, and sulfur atoms are painted white, blue, red, and yellow, respectively. A green wire representation is adopted for H-bonds.

The homology model of the rat L-type voltage-gated calcium channel Ca_V_1.2 (rCa_V_1.2) α_1C_ subunit was built using as a template the cryo-EM structure of the human ortholog (PDB id: 8EOG), with which it shares >97% of sequence identity. The model so obtained was used for the subsequent docking studies of the methoxyflavones, i.e., tangeretin, scutellarein TME, sinensetin, and nobiletin at the fenestration site between the repeats S5_III_ and S6_IV_ [21]. As shown in Figure 7, the representative docking poses for the most active compounds, i.e., scutellarein TME and tangeretin, selected based on the most favorable docking score values, were very similar: in both cases, the *p*-methoxy-phenyl ring was sandwiched between Trp1447 and Tyr1489 and engaged a π-π stacking with the latter. Steric hindrance of the additional methoxy group on the pendant phenyl ring (see Figure S8, SI) precluded both sinensetin and nobiletin from reaching similar arrangements. This difference could explain the lower activity of these compounds compared with their monomethylated counterparts. The carbonyl group of the bicyclic ring of both scutellarein TME and tangeretin forms an H-bond with Ser1141 side chain, while C5- and C6- methoxy groups are engaged in H-bonds with Thr1065 and Gln1069 side chains, respectively, thus recapitulating the polar interactions observed in the Ca_V_1.1-nifedipine complex.

## 3. Discussion

The findings of the present study demonstrate how a multidisciplinary approach can isolate novel potential vasoactive agents from an agricultural by-product such as citrus fruit peels. This can have at least two important outcomes: (1) the future exploitation of food waste otherwise representing an economic burden for society and a hazard for the environment; (2) additional weapons in the arsenal of cardiovascular drugs.

In the first step of the study, the sequential use of different extraction methods from blond orange peels bioguided by an in vitro vascular activity assessment led to the separation of fractions M3 and M4, which showed an interesting spasmolytic effect on pre-contracted aorta rings. The following step, consisting of the chemical characterization of both fractions, performed by NMR, HRMS, and UPLC-HRMS/MS, identified the single components likely responsible for this biological activity. The presence of scutellarein TME, nobiletin, tangeretin, and sinensetin in fraction M3 and only sinensetin in fraction M4 was confirmed by comparison with the literature data and standards spectra acquisition in-house, as well as by MCR of NMR fraction spectra. Therefore, the combination of NMR datasets with the MCR-ALS algorithm provided a powerful tool for unraveling complex metabolic data and allowed for a satisfactory resolution of individual peaks in the NMR signals, leading to the identification of different metabolites through the comparison of calculated spectra with those recorded from the standards.

The in vitro biological analysis of the extract, fractions, and their single components, demonstrated that a proper extraction methodology can provide effective vasorelaxant agents from a food by-product such as blond orange peels. Furthermore, the results highlighted a higher efficacy and potency against phenylephrine- than against KCl-induced contraction. Both phenomena cause a rise in cytoplasmic Ca^2+^ concentration that triggers muscle contraction. However, during phenylephrine-induced contraction, Ca^2+^ release from the intracellular stores and extracellular Ca^2+^ influx through Ca_V_1.2, receptor-operated, and store-operated Ca^2+^ channels occur whereas KCl-induced contraction essentially depends on extracellular Ca^2+^ influx through Ca_V_1.2 channels [21]. Therefore, it is conceivable to hypothesize that extracts, fractions, and their single components affect at least one of these pathways to cause vasorelaxation. One possible target might be the Ca_V_1.2 channel as both the DCM phase and M3 fraction relaxed the contraction induced by high extracellular KCl concentrations that cause membrane depolarization and Ca_V_1.2 channel opening (electromechanical coupling). Indeed, the patch-clamp data, recorded under experimental conditions mimicking those of the functional experiments, provided direct evidence that the M3 fraction contains effective Ca_V_1.2 channel blockers, namely, scutellarein TME, tangeretin, nobiletin, and sinensetin. In fact, both the M3 fraction and the four PMFs antagonized I_Ba1.2_ in a concentration-dependent manner. Furthermore, as these data were collected under the whole-cell configuration, which causes extensive dialysis of the cytoplasm and the second messengers therein, it is conceivable to hypothesize that the Ca_V_1.2 channel blockade is due to a direct interaction of the PMFs with the channel protein. This hypothesis is substantiated by the in silico analysis, as detailed below.

To the best of our knowledge, this is the first direct evidence of the Ca_V_1.2 channel-blocking activity of PMFs present in citrus waste. Furthermore, it is worth noting how methylation of the four OH groups of scutellarein transformed the molecule from a Ca_V_1.2 channel stimulator [22] into a blocker, once more supporting the importance of PMFs as a novel class of calcium antagonists. Finally, tangeretin, sinensetin, and nobiletin significantly accelerated the inactivation kinetics of I_Ba1.2_, thus suggesting they favored the transition from the open to the inactivated state of the channel. Whether these PMFs are also capable of stabilizing the inactivated state of the Ca_V_1.2 channel, as suggested by the in silico analysis for scutellarein TME and tangeretin (see below), remains to be elucidated.

The higher vasorelaxant efficacy and potency (except M2 fraction) recorded in endothelium-deprived preparations pre-contracted by phenylephrine compared with those stimulated by KCl, suggested that these agents modulate additional target(s) located either on the plasmalemma or on the sarcoplasmic reticulum. Furthermore, the endothelium proved to be the target responsible for their vasoactivity: its removal significantly affected the potency of both E and DCM phases, scutellarein TME and tangeretin, and partially reduced that of nobiletin, as previously observed by Kaneda et al. [23]. These results indicate that the vasorelaxant activity of scutellarein TME and tangeretin is, at least partially, endothelium-dependent and likely underpins that observed with the DCM phase. This phenomenon is commonly ascribed to the release of vasoactive agents such as NO, prostacyclin, or endothelium-derived hyperpolarizing factor. Investigation of the factor involved, however, goes beyond the scope of the present study. It is worth noting that the endothelium-dependent vasorelaxation disappeared in the four fractions, which showed similar behaviors both in the presence or absence of a functional endothelium. This is somewhat surprising, at least for the M3 fraction containing the endothelium-dependent vasodilators scutellarein TME, tangeretin, and nobiletin. At present, we have no explanation for this phenomenon; we can only speculate that the effect of the endothelium-independent vasodilator sinensetin prevails hierarchically over that of the other three major PMFs present in the fraction.

As stated above, the M4 fraction contains only sinensetin. The biological data further support this evidence: in fact, pure sinensetin displayed potency and efficacy values against phenylephrine- and KCl-induced contraction that were like those recorded with the M4 fraction.

To get an insight into the binding modes of the compounds investigated, a docking study was carried out using a homology model of the rat Ca_V_1.2 channel. The α_1_ subunit of Ca_V_ channels folds in four homologous repeats I–IV. Each repeat comprises six transmembrane segments (S1–S6) arranged in the canonical voltage-gated ion channel fold. The recently solved cryo-EM structures of the Ca_V_1.1 channel, which belongs to the same subfamily of the Ca_V_1.2 channel, in complex with some modulators, disclosed distinct binding sites for nifedipine and the positively charged blockers verapamil and diltiazem. While the binding site for the latter is in the central cavity of the pore domain where they act as channel blockers, the nifedipine binding site is located at the interface of repeats S5_III_ and S6_IV_ [24]. Due to the neutral charge of the investigated methoxyflavones, docking studies were focused on the nifedipine binding site. The findings of the present study showed that only the monomethoxylate phenyl pendant, featured by both scutellarein TME and tangeretin, could properly interact with Trp1447 and Tyr1489 residues on S6_IV_, bringing together the two repeats of the fenestration site and, in turn, stabilizing the inactivated state of the channel. In fact, the same arrangement was precluded for the less active dimethoxylate compounds sinensetin and nobiletin due to steric hindrance. Looking in further detail at the docking poses of scutellarein TME and tangeretin, the additional C8-methoxy group occurring in the tangeretin bicyclic scaffold induced the displacement of the Met1490 sidechain, thus supporting the slightly better activity of scutellarein TME compared with tangeretin.

Though the electrophysiology and molecular modelling, evidence strongly supports the hypothesis that the spasmolytic activity of PMFs observed on depolarised rings is due to the blockade of Ca_V_1.2 channels, and the effects on Ca^2+^ sensitivity of the contractile proteins cannot be ruled out. Furthermore, other targets, such as cyclic nucleotide-dependent pathways, can be involved in their spasmolytic activity, as previously observed for nobiletin [23], sudachitin, and demethoxysudachitin [25]. Interestingly, the four polymethoxyflavones analyzed possess also an effective anti-inflammatory activity observed in several model systems (see for example [1,26,27,28,29]). This activity, along with the Ca^2+^ antagonistic activity described here, makes them a valuable starting point for developing double-edged swords for treating cardiovascular diseases.

So far, studies performed on human or rodent models have investigated mainly the anti-inflammatory and antitumor effects of polymethoxyflavones; those addressing the cardiovascular effects, however, are scarce or even missing. Li et al. [30] have demonstrated that total phenolic extracts of *Citrus aurantium* L. protect rats from hyperhomocysteinemia-induced vascular endothelial dysfunction modulating arachidonic acid metabolism and reducing oxidative stress. Therefore, it is now necessary to verify whether the present results can be translated into animal models and, hopefully, into human models, thus consolidating the concept that citrus peel is, indeed, a novel source of cardiovascular protecting agents.

## 4. Materials and Methods

### 4.1. Chemicals and Instruments

Organic solvents (ACS grade) for extraction and column chromatography separation were purchased from Merck (Merck KGaA, Darmstadt, Germany) and used as received. Ultrasound-assisted extraction was performed using an ultrasonic water bath Grant XUB5 digital control. Flash column chromatography was carried out on silica gel (Merck KGaA, Darmstadt, Germany, Kieselgel 60, particle size 0.040–0.063 mm). Thin-layer chromatography (TLC) was carried out on Merck 0.2 mm precoated silica (60 F254) aluminum sheets, with visualization by irradiation with a UV lamp (254 and 365 nm). ^1^H NMR spectra were recorded at 400 MHz using a Bruker Avance 400 MHz spectrometer. MS-grade solvents used for LC-MS analysis, namely, acetonitrile (MeCN), methanol (MeOH), water (H_2_O), and formic acid (HCOOH) were provided by Romil (Cambridge, UK). Sinensetin, nobiletin, scutellarein tetramethyl ether, and tangeretin standards were from MedChemtronica AB. For LC-MS analysis, the four methoxyflavones standards, as well as the isolated fractions, were dissolved in methanol.

A UPLC separation system consisting of a quaternary Accela 600 pump and an Accela autosampler coupled to a high-resolution mass spectrometry (LC-ESI/HRMS, ThermoScientific, San Jose, CA, USA) LTQ Orbitrap XL mass spectrometer (ThermoScientific, San Jose, CA, USA), operating in positive electrospray ionization mode, was used for qualitative analysis. Data were collected and analyzed using the Xcalibur 2.2 software provided by the manufacturer. The separation was carried out using a Luna 2.5 u C18 (2)-HST reverse phase column (100 × 2.0 mm) equipped with a security guard HPLC Guard Cartridge System (Phenomenex, Castel Maggiore, Bologna, Italy). The flow rate was set at 300 µL/min, solvent A was water, and solvent B was MeCN, both containing 0.1% formic acid (*v*/*v*). The chromatographic system was operated in gradient mode as follows: initial B concentration was 20% and kept constant for 5 min. The B concentration was linearly increased up to 80% in 10 min, then further increased to 99% in 1 min, and maintained in this condition for 2 min of column washing. A column equilibrium time of 7 min was set before the following injection. The column temperature was set at 35 °C and the autosampler was set to inject 2 μL of sample. In the positive ion mode, the following experimental conditions for the ESI source were adopted: sheath gas at 15 (arbitrary units), auxiliary gas at 10 (arbitrary units), source voltage at 4.5 kV, capillary temperature at 280 °C, capillary voltage at 27 V, and tube lens at 105 V. The mass range was from 100 to 500 *m*/*z* with a resolution of 30,000. Tandem mass (MS/MS) product ions were obtained with a normalization collision energy at 30%, a minimum signal threshold of 250, and an isolation width of 1.0. For quantitative purposes, the separation was performed using the same UPLC column and chromatographic conditions on an Acquity UPLC system (Waters GmbH; Eschborn, Germany) equipped with both a photodiode array (PDA) detector and a QDa mass detector. The UV detector was collected in the range of 190–400 nm. The QDa MS detector was operated in positive ESI ionization. For each analyte, a five-point calibration point was obtained in the range from 0.1 to 50 ng.

The chemicals used in the biological assays included collagenase (type XI), trypsin inhibitor, bovine serum albumin, TEA chloride, phenylephrine, acetylcholine, nifedipine (Sigma Chimica, Milan, Italy), tangeretin and scutellarein TME (Extrasynthese; Genay Cedex, France), nobiletin and sinensetin (DBA, Segrate, Milan, Italy), and sodium nitroprusside (Riedel-De Haen AG, Seelze Hannover, Germany). All remaining substances were of analytical grade and used without additional purification. Phenylephrine was dissolved in 0.1 M HCl, while sodium nitroprusside was dissolved in distilled water. Nifedipine, directly dissolved in ethanol, and the analyzed compounds, directly dissolved in DMSO, were diluted at least 1000 times before use.

Control experiments were conducted to validate the absence of any vascular response when DMSO or ethanol was added alone at their respective final maximal concentration of 0.1% (*v*/*v*).

### 4.2. Extraction

Orange peels were obtained from fresh commercially mature blond oranges. Fruits were washed with water, and peels were separated by hand from the pulp and cut into small pieces. Then ethanol 70% in water solution (150 mL/28 g of peels) was added, and the resulting mixture was sonicated at 35 °C for 1 h. The hydroalcoholic extract (50 mL) was first washed with *n*-hexane (3 × 50 mL) and then with dichloromethane (3 × 50 mL). Combined dichloromethane extracts were evaporated under reduced pressure, and the resulting residue was purified by flash column chromatography (SiO_2_) using a mixture of CHCl_3_/Et_2_O (6:4) as an eluent to give M1–M4 fractions.

### 4.3. Biological Assays

#### 4.3.1. Animals

All study procedures strictly adhered to the European Union Guidelines for the Care and the Use of Laboratory Animals (European Union Directive 2010/63/EU) and received approval from the Animal Care and Ethics Committee of the University of Siena and the Italian Department of Health (7DF19.N.SJQ on the 12 April 2024). Male Wistar rats (260–360 g), purchased from Charles River Italia (Calco, Italy), were housed in an animal facility with a temperature of 25 ± 1 °C and a 12:12 h dark–light cycle. They were fed a standardized diet and had access to drinking water ad libitum. Animals were anesthetized with isoflurane (4%)-O_2_ gas mixture using Fluovac equipment (Harvard Apparatus, Holliston, MA, USA), followed by decapitation and exsanguination. The thoracic aorta and tail main artery were promptly isolated and placed in a modified Krebs-Henseleit solution (KHS) or an external solution (see below for composition), respectively. Cells and rings were processed as detailed in the following sections.

#### 4.3.2. Aorta Ring Preparation and Functional Experiments

The thoracic aorta was gently cleaned of adipose and connective tissues and sectioned into 3–4-mm wide rings. These were mounted in organ baths between two parallel, L-shaped, stainless-steel hooks, one fixed in place and the other connected to an isometric transducer [31] (BLPR, WPI, Berlin, Germany). Rings equilibrated for 60 min in KHS [composition (in mM): 118 NaCl, 4.75 KCl, 1.19 KH_2_PO_4_, 1.19 MgSO_4_, 25 NaHCO_3_, 11.5 glucose, 2.5 CaCl_2_, gassed with a 95% O_2_–5% CO_2_ gas mixture to create a pH of 7.4] under a passive tension of 1 g based on preliminary experiments where higher or lower passive tensions gave rise to unstable and not sustained contractions. Throughout this equilibration period, the solution was replaced every 15 min. Isometric tension was recorded using a digital PowerLab data acquisition system (PowerLab 8/30; ADInstruments, Castle Hill, Australia). The viability of the rings was assessed by recording the response to 0.3 µM phenylephrine and 60 mM KCl. Where needed, the endothelium was removed by gently rubbing the lumen of the ring with a forceps tip. This procedure was validated by adding 10 µM acetylcholine at the plateau of phenylephrine-induced contraction: a relaxation ≥75% or <15% denoted the presence or absence of functional endothelium, respectively [32].

Extracts and pure compounds were assessed on rings pre-contracted by either 25 mM or 60 mM KCl and by 0.3/0.6 µM phenylephrine, respectively.

#### 4.3.3. Cell Isolation Procedure for Patch-Clamp Experiments

Smooth muscle cells were freshly isolated from the tail main artery, at 37 °C, using collagenase (type XI, 1.2 mg/mL) treatment in the presence of bovine serum albumin (1 mg/mL) and trypsin inhibitor (1 mg/mL) in 2 mL of 0.1 mM Ca^2+^ external solution [consisting of (in mM): 130 NaCl, 5.6 KCl, 10 HEPES, 20 glucose, 1.2 MgCl_2_, and 5 Na-pyruvate; pH 7.4] containing 20 mM taurine. The isolation process was conducted under a gentle stream of a 95% O_2_–5% CO_2_ gas mixture as previously described [33]. Cells, stored in a 0.05 mM Ca^2+^ external solution containing 20 mM taurine and 0.5 mg/mL bovine serum albumin at 4 °C under normal air, were used for experiments within two days after isolation [17].

#### 4.3.4. Ba^2+^ Current Through Ca_V_1.2 Channel (I_Ba1.2_) Recordings

The conventional whole-cell configuration of the patch-clamp technique was applied to record I_Ba1.2_ from freshly isolated vascular smooth muscle cells. Borosilicate recording electrodes (WPI, Berlin, Germany) were used, having a pipette resistance of 2–4 MΩ when filled with the internal solution [containing (in mM): 100 CsCl, 10 HEPES, 11 EGTA, 1 CaCl_2_ (pCa 8.4), 2 MgCl_2_·6 H_2_O, 5 Na-pyruvate, 5 succinic acid, 5 oxaloacetic acid, 3 Na_2_-ATP, and 5 phosphocreatine; pH was adjusted to 7.4 with CsOH]. An Axopatch 200B patch-clamp amplifier (Molecular Devices Corporation, Sunnyvale, CA, USA) was used with an ADC/DAC interface (DigiData 1200 A/B series, Molecular Devices Corporation). The setup facilitated the generation and application of voltage pulses, recording of corresponding membrane currents, adjustment to zero of the junction potential between the pipette and bath solution, and compensation of whole-cell capacitance and series resistance (between 70–75%). Cells where the leak current was greater than 20% of the initial I_Ba1.2_ were discarded. Current signals underwent low-pass filtering at 1 kHz and were digitized at 3 kHz before being stored on the computer hard disk. Cells were continuously superfused at room temperature (20–22 °C) with an external solution containing 0.1 mM Ca^2+^ and 30 mM tetraethylammonium (TEA^+^) using a peristaltic pump (LKB 2132, Bromma, Sweden), at a flow rate of 400 µL/min.

I_Ba1.2_ was recorded in an external solution containing 30 mM TEA^+^ and 5 mM Ba^2+^. The current was elicited with 250-ms clamp pulses (0.067 Hz) to 10 mV from a holding potential (V_h_) of −50 mV. Whole-cell capacitance and series resistance were checked and compensated every 5 min of recordings or before if necessary. Data were collected once the current amplitude had stabilized (typically 7–10 min after achieving the whole-cell configuration). Under these conditions, I_Ba1.2_ remained stable for the subsequent 30–40 min [34].

K^+^ currents were blocked with 30 mM TEA^+^ in the external solution and Cs^+^ in the internal solution. Current values were corrected offline for leakage and residual outward currents using 10 µM nifedipine, which completely blocked I_Ba1.2_.

### 4.4. Statistical Analysis

Individual values contributing to the calculation of the group mean ± SD were derived from independent cells or rings (n). These cells or rings, although occasionally isolated from the same animal (with at least three different animals in the same group), were randomly assigned to different treatments and assessed individually, thereby considered as single experimental units. Data were normally distributed, and variances were not significantly different.

Data analysis was conducted using pClamp 9.2.1.8 software (Molecular Devices Corporation), LabChart 7.3.7 Pro (PowerLab; ADInstruments), and GraphPad Prism version 5.04 (GraphPad Software Inc., San Diego, CA, USA). Statistical analyses along with significance determination through Student’s *t*-test, paired or unpaired, and one-way ANOVA were performed using GraphPad Prism version 5.04. In all comparisons, *p* < 0.05 was considered significant. The pharmacological response to drugs was expressed in terms of potency (pIC_50_ or IC_50_ values, i.e., the negative logarithm of or the drug concentration causing a reduction of the biological signal equal to 50% of the maximum, obtained through nonlinear regression analysis) and efficacy (E_max_ value, i.e., the maximum effect evoked by the drug). Concentration–response curves with data not sufficient to allow for pIC_50_ or IC_50_ and E_max_ value calculations were analyzed with two-way ANOVA and the Bonferroni multiple comparison test.

### 4.5. Computational Method

Starting ligand geometries were retrieved from PubChem (https://pubchem.ncbi.nlm.nih.gov/, accessed on 24 November 2023;) database (CID: 145659, 72344, 68077, and 96118 for sinensetin, nobiletin, tangeretin, and scutellarein TME, respectively). The molecules were fully optimized using the GAMESS program 2021 R1 [35] at the Hartree–Fock level with the STO-3G basis set and subjected to HF/6-31G*/STO-3G single-point calculations to derive the partial atomic charges using the RESP procedure [36]. Docking studies were performed with AutoDock 4.2 [37], using a homology model of rat rCa_V_1.2 α_1c_ subunit, built using the available X-ray structure of the human orthologue (PDB id: 8EOG) as a template with the MODELLER v10.1 program [38]. Ten homology models were built using the alignment obtained using clustalw. The best model in terms of both the Modeller objective function and the Dope score was selected for the subsequent docking calculations. Docking runs were carried out by allowing the rotation of selected residues, namely, Gln1069, Phe1138, Ile1486, and Met1490. Both proteins and ligands were processed with AutoDock Tools (ADT) package version 1.5.7 [37] to merge nonpolar hydrogens and calculate Gasteiger charges. Grids for docking evaluation, with a spacing of 0.375 Å and 60 × 50 × 50 points centered on the nifedipine binding site, were generated using the program AutoGrid 4.2 included in Autodock 4.2 distribution following a previously published docking protocol [39]. The complexes, selected based on binding energy and cluster population, were completed by the addition of all hydrogen atoms and underwent energy minimization with Amber 20 package [40], using ff14SB force (proteins), and gaff2 (ligand) force field parametrization. UCSF Chimera 1.17 [41] was used for visualization and figures.

### 4.6. Multivariate Curve Resolution (MCR)

To identify the compounds in the extraction procedure, the raw ^1^H-NMR spectra of the four standard compounds and fraction M3 (all samples in triplicate) were exported and converted into human-readable files (.csv), ready for direct import into MATLAB R2023a^®^ (The MathWorks, Inc., Natick, MA, USA). All chemometric analyses were conducted within the MATLAB^®^ computing environment, utilizing the *MCR-ALS routines* (GUI version 2.0) [42]. The uninformative regions at the extremes of the spectrum, above 9 ppm, below 3 ppm, and at negative ppm values, where no signals of appreciable intensity were observed were removed from the spectral data. This process resulted in a dataset containing 14,058 experimental data points. Subsequently, the data were downsampled for computational convenience and organized into a numerical matrix with dimensions of 15 × 7029 (samples × variables). The pre-treatment of data is a critical step in any multivariate data analysis workflow and is highly dependent on the specific case. For NMR data, an alignment procedure is necessary before any subsequent data handling. Spectra alignment was conducted using the *icoshift* algorithm [43]. Signal alignment is essential because even minor variations in experimental conditions can lead to horizontal shifts in peaks, which, although minimized, may introduce variability not attributable to actual differences among the samples. Alignment was conducted by applying *icoshift* to the spectra using the average spectrum as a reference and dividing the spectra into 50 regularly spaced intervals. The resulting aligned dataset was then utilized as input for the multivariate data analysis.

## 5. Conclusions

Calcium channel blockers play a pivotal role in the management of cardiovascular diseases, the leading cause of death worldwide [44]. The continuously growing global incidence of these diseases, with or without comorbidity, requires the development of novel Ca_V_1.2 channel blockers with fewer side effects than those associated with dihydropyridines, phenylalkylamines, and benzothiazepines, currently administered in therapy, which limit the compliance of patients. Although several in vitro and in vivo studies evidenced different mechanisms underpinning the beneficial effects ascribed to PMFs against many diseases including cardiovascular diseases, this is the first time that Ca_V_1.2 channel antagonism has been reported. Molecular modeling studies provided a structural basis to rationalize the in vitro inhibitory effect of the four isolated *Citrus* peel PMFs on I_Ba1.2_. The resultant SAR will undoubtedly inspire the development of novel Ca_V_1.2 channel blockers based on the flavone backbone.

## Figures and Tables

**Table 1:** Vasorelaxant potency and efficacy of extracts and fractions on either phenylephrine- or KCl-induced contraction.

Sample	Phenylephrine + Endothelium		Phenylephrine − Endothelium		KCl	
	IC_50_ (µg/mL)	E_max_ (%)	IC_50_ (µg/mL)	E_max_ (%)	IC_50_ (µg/mL)	E_max_ (%)
E	31.72 ± 18.04 (5)	74.3 ± 7.2 (5)	≈100	50.8 ± 15.6 * (5)	N.D.	14.0 ± 7.9 ^§^ (5)
DCM	8.46 ± 4.68 ^†^ (5)	97.1 ± 3.3 ^†^ (4)	52.19 ± 29.51 * (5)	70.9 ± 14.6 * (5)	N.D.	22.1 ± 22.6 ^§^ (5)
**M1**	13.56 ± 9.54 (4)	92.7 ± 4.6 (4)	21.45 ± 7.05 (5)	93.1 ± 4.9 (5)	N.D.	41.9 ± 6.3 ^§^ (5)
**M2**	N.D.	43.6 ± 17.1 (4)	N.D.	29.9 ± 13.6 (3)	N.D.	33.7 (2)
**M3**	0.45 ± 0.14 (5)	97.8 ± 1.6 (5)	0.68 ± 0.46 (6)	96.5 ± 2.8 (6)	5.22 ^#^	73.9 ± 21.4 ^§^ (5)
**M4**	2.99 (2)	100 (1)	5.40 ± 3.94 (3)	100 (1)	49.40 ^#^	67.0 ± 13.0 (3)

Potency (IC_50_) and efficacy (E_max_) values are reported as mean ± SD. Replicates are shown in brackets. E: *n*-hexane; DCM: dichloromethane; N.D.: not detected. ^#^ Estimated value. * *p* < 0.05 vs. +endothelium, ^†^ *p* < 0.05 vs. E, ^§^ *p* < 0.05 vs. phenylephrine − endothelium, Student’s *t*-test for unpaired samples (two-tailed).

## Data Availability

The datasets generated and/or analyzed during the current study are available from the corresponding author upon reasonable request.

## Supplementary Materials

The following supporting information can be downloaded at: https://www.mdpi.com/article/10.3390/molecules29235693/s1. HRMS spectrum of M3 fraction: Figure S1. ^1^H NMR (400 MHz, CDCl_3_) of M3: Figure S2. ^1^H NMR (400 MHz, CDCl_3_) of M3 in the range 6.3–8.5 ppm: Figure S3. ^1^H NMR (400 MHz, CDCl_3_) of M3 in the range 3.8–4.3 ppm: Figure S4. ^1^H NMR (400 MHz, CDCl_3_) of M4: Figure S5. LC-HRMS and LC-HRMS/MS spectra of the four major components of the M3 fraction: Figure S6. HRMS (ESI) *m*/*z* [M + H]^+^ calculated and found for compounds reported in Figure S6: Table S1. In source ESI-CID spectra of four major components of the M3 mixture compared with separated analytical standards: Figure S7. Representative docking poses for nobiletin and sinensetin: Figure S8.
