# Supplementary material for: Bioguided Identification of Polymethoxyflavones as Novel Vascular CaV1.2 Channel Blockers from Citrus Peel
Source: Molecules. 2024 Dec 2;29(23):5693. doi: 10.3390/molecules29235693 (PMC11642981; doi:10.3390/molecules29235693)
Supplement: Supplementary file 1 [file molecules-29-05693-s001.zip › molecules-3284957-supplementary.pdf]

Supplementary Material

Bioguided identification of polymethoxyflavones as novel vascular $Ca_V1.2$ channel blockers

*Anna Ramunno[a], Rosa Maria Vitale[b], Pietro Amodeo[b], Carlo Crescenzi[a], Alice Panti[c], Paolo Fiorenzani[d], Michele De Luca[e], Umile Gianfranco Spizzirri[f], Donatella Restuccia[g], Francesca Aiello[*e], and Fabio Fusi[d]*

Table of content

HRMS spectrum of **M3** fraction: **Figure S1**

^{1}H NMR (400 MHz, $CDCl_3$) of **M3: Figure S2**

^{1}H NMR (400 MHz, $CDCl_3$) of **M3** in the range 6.3-8.5 ppm: **Figure S3**

^{1}H NMR (400 MHz, $CDCl_3$) of **M3** in the range 3.8-4.3 ppm: **Figure S4**

^{1}H NMR (400 MHz, $CDCl_3$) of **M4: Figure S5**

LC-HRMS and LC-HRMS/MS spectra of the four major components of the **M3** fraction: **Figure S6**

HRMS (ESI) *m/z* $[M+H]^+$ calculated and found for compounds reported in Figure S6: **Table S1**

In source ESI-CID spectra of four major component of the **M3** mixture and separated analytical standards: **Figure S7**

Representative docking poses of nobiletin: **Figure S8**

Figure S1: HRMS spectrum of **M3** fraction : **A**) range 200-900 and **B**) range 300-500

A)

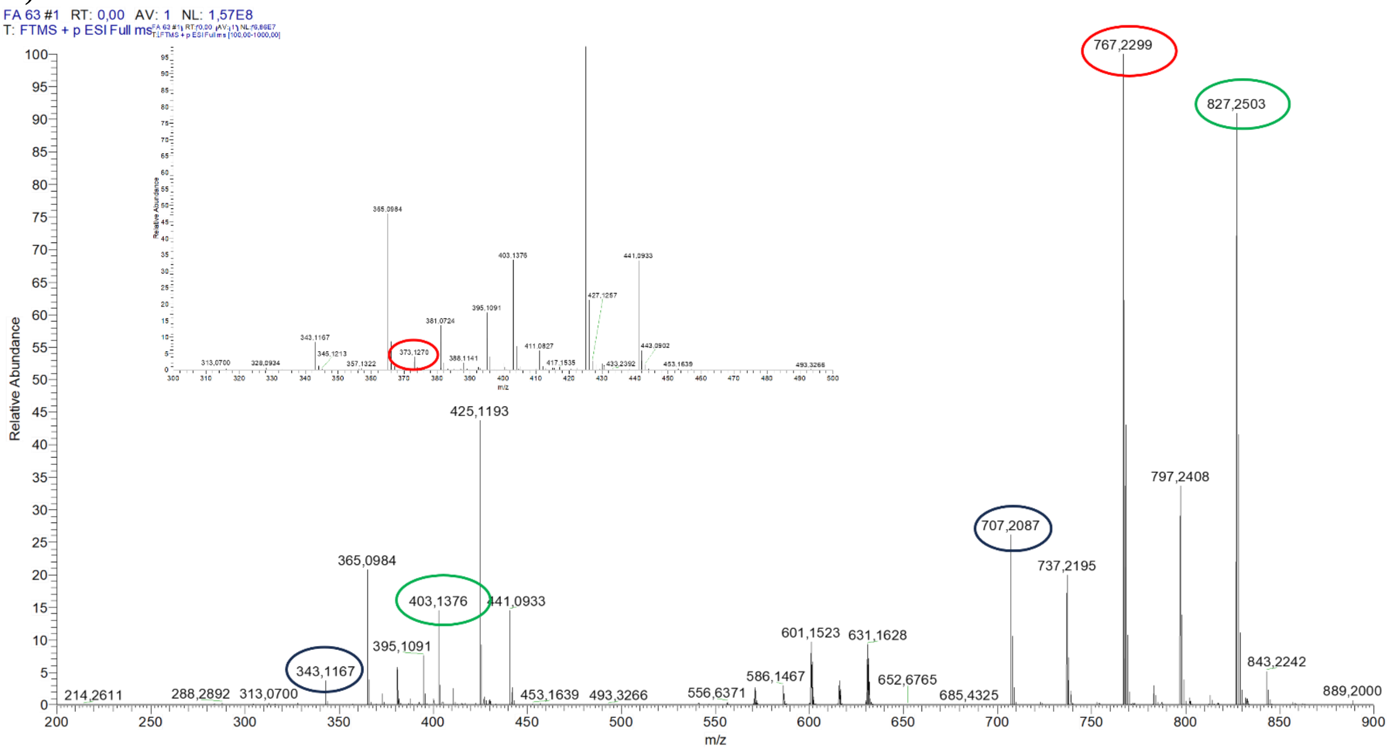

B)

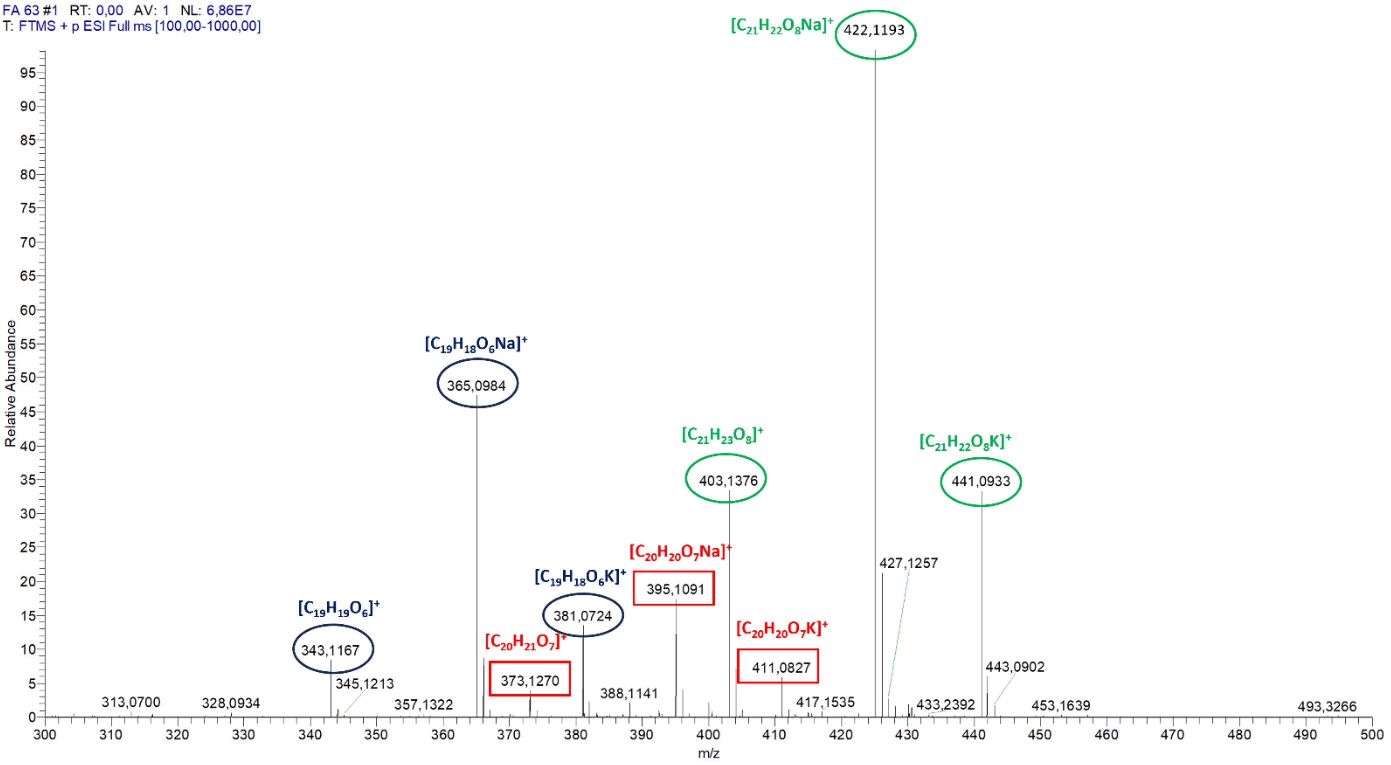

Figure S2: ^{1}H NMR (400 MHz, $CDCl_3$) of **M3**

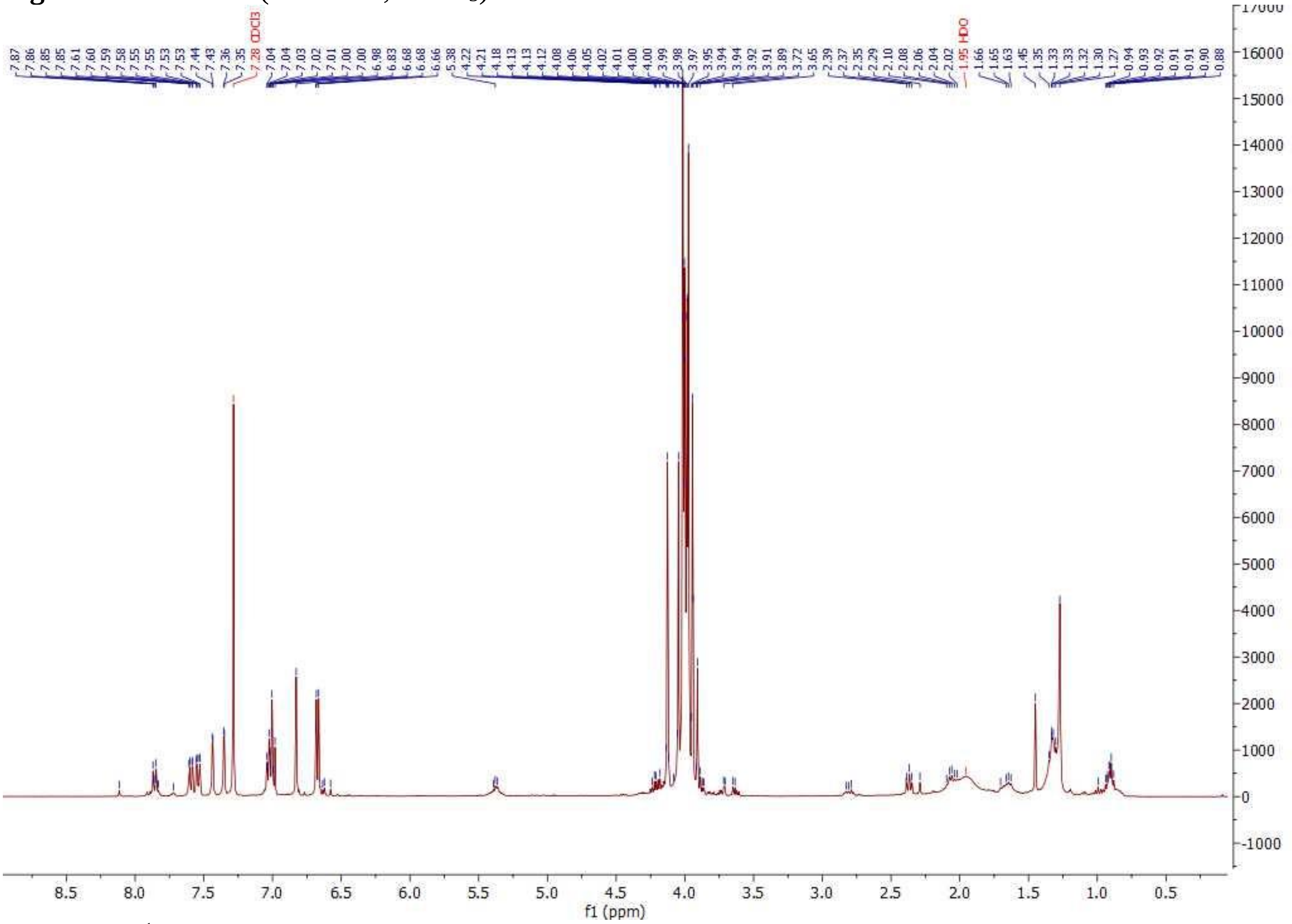

Figure S3: ^{1}H NMR (400 MHz, $CDCl_3$) of **M3** in the range 6.3-8.5 ppm.

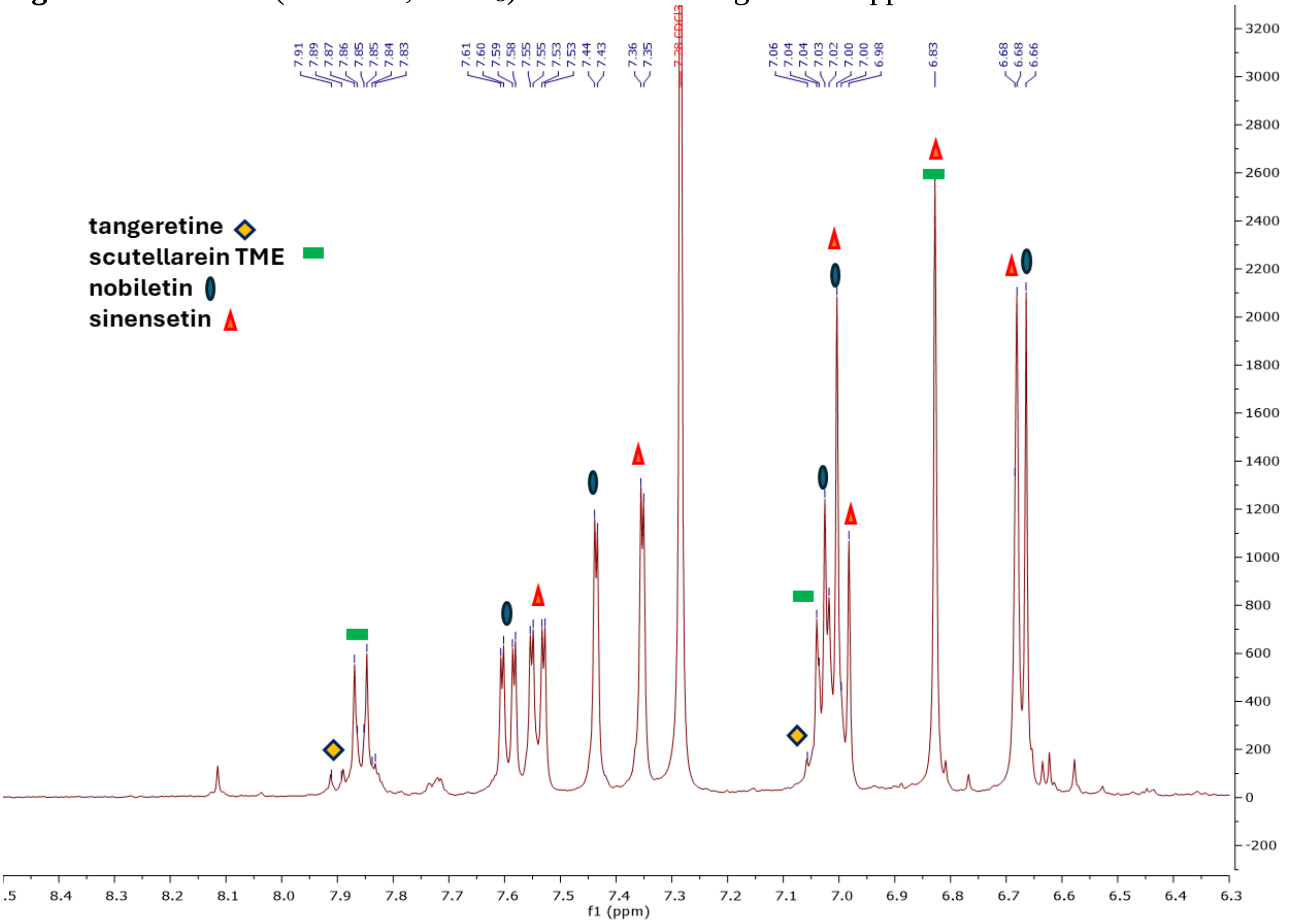

Figure S4: ^{1}H NMR (400 MHz, $CDCl_3$) of **M3** in the range 3.8-4.3 ppm

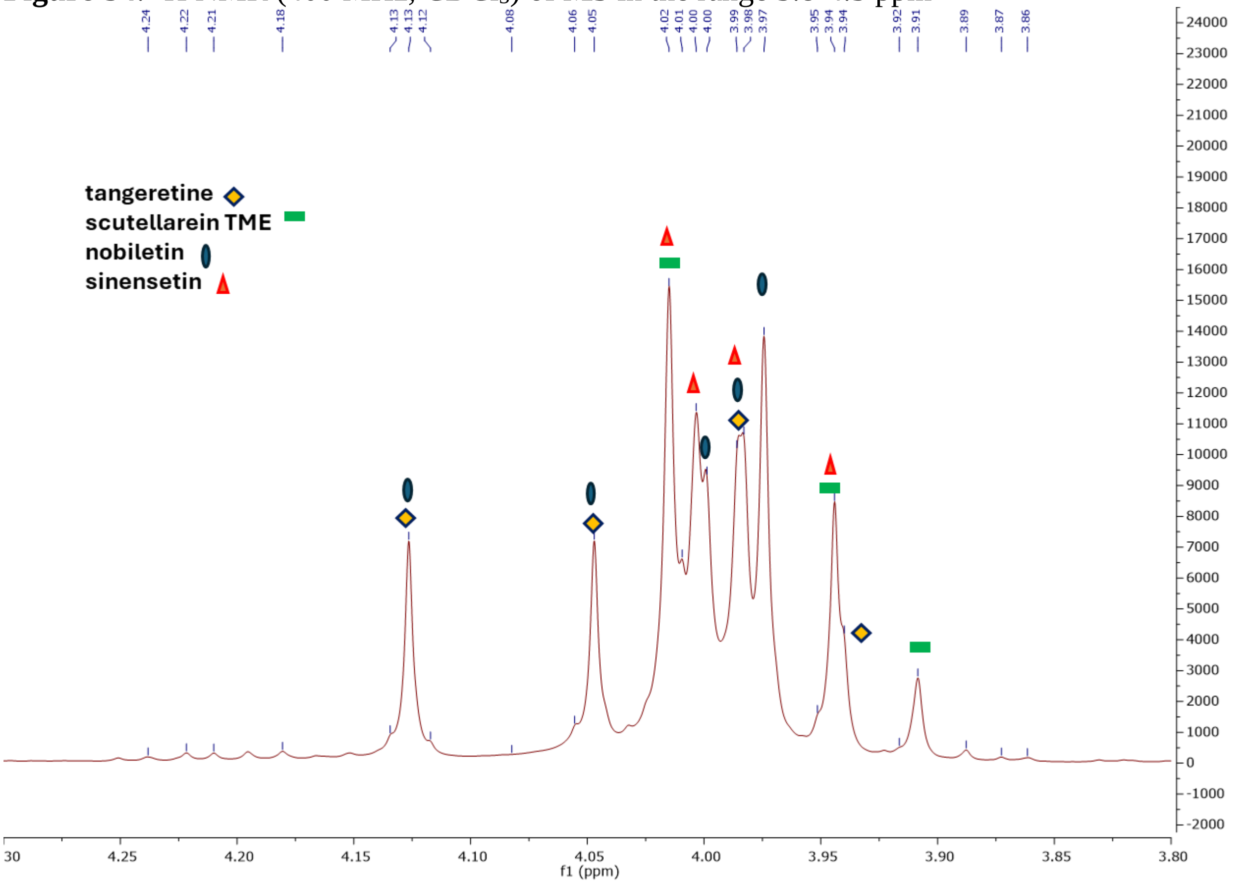

Figure S5: ^{1}H NMR (400 MHz, $CDCl_3$) of **M4**

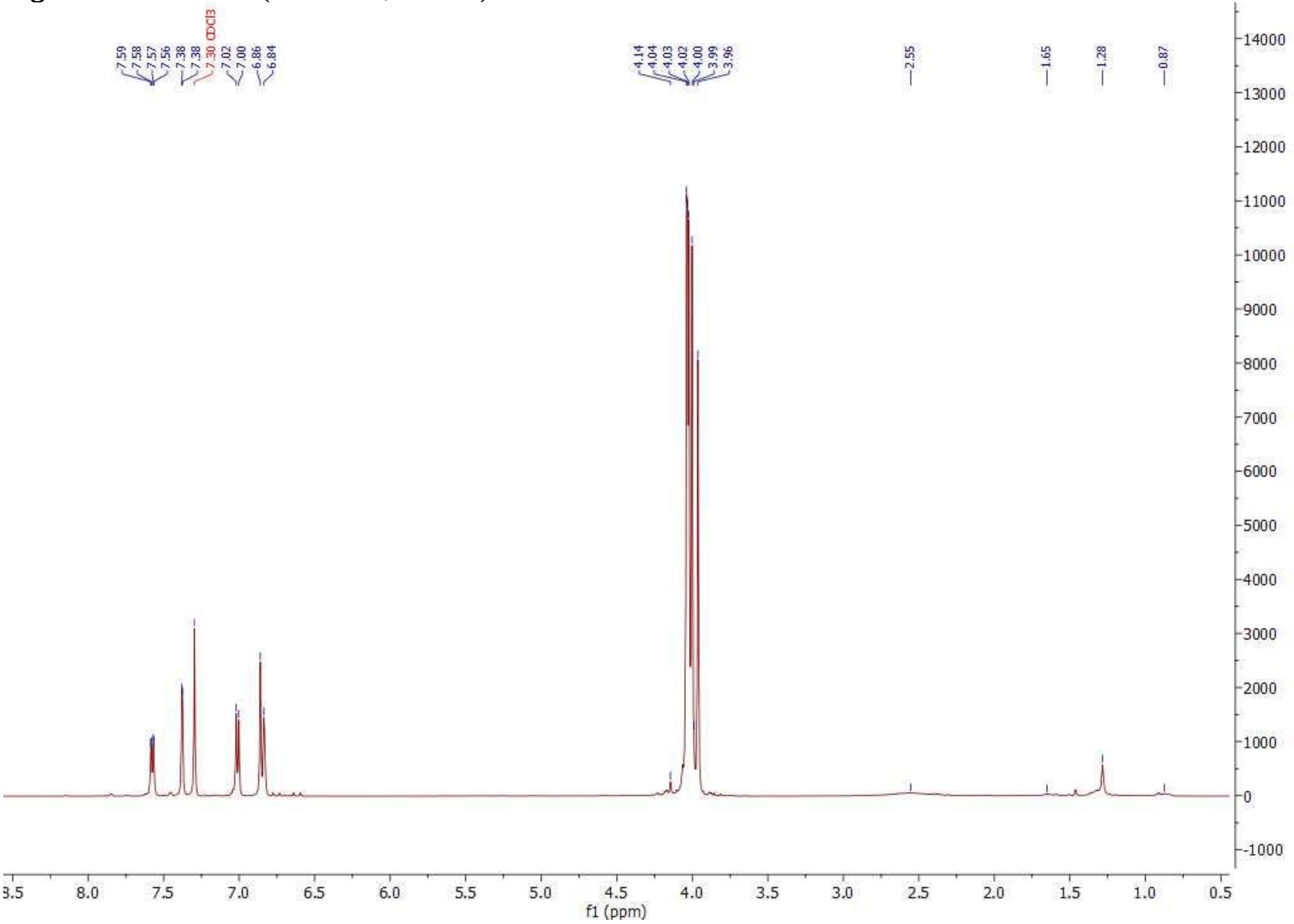

Figure S6: LC-HRMS and LC-HRMS/MS spectra of the four major components of the **M3** fraction: A) sinensetin, B) nobiletin, C) scutellareine tetramethyl ether, D) tangeretin

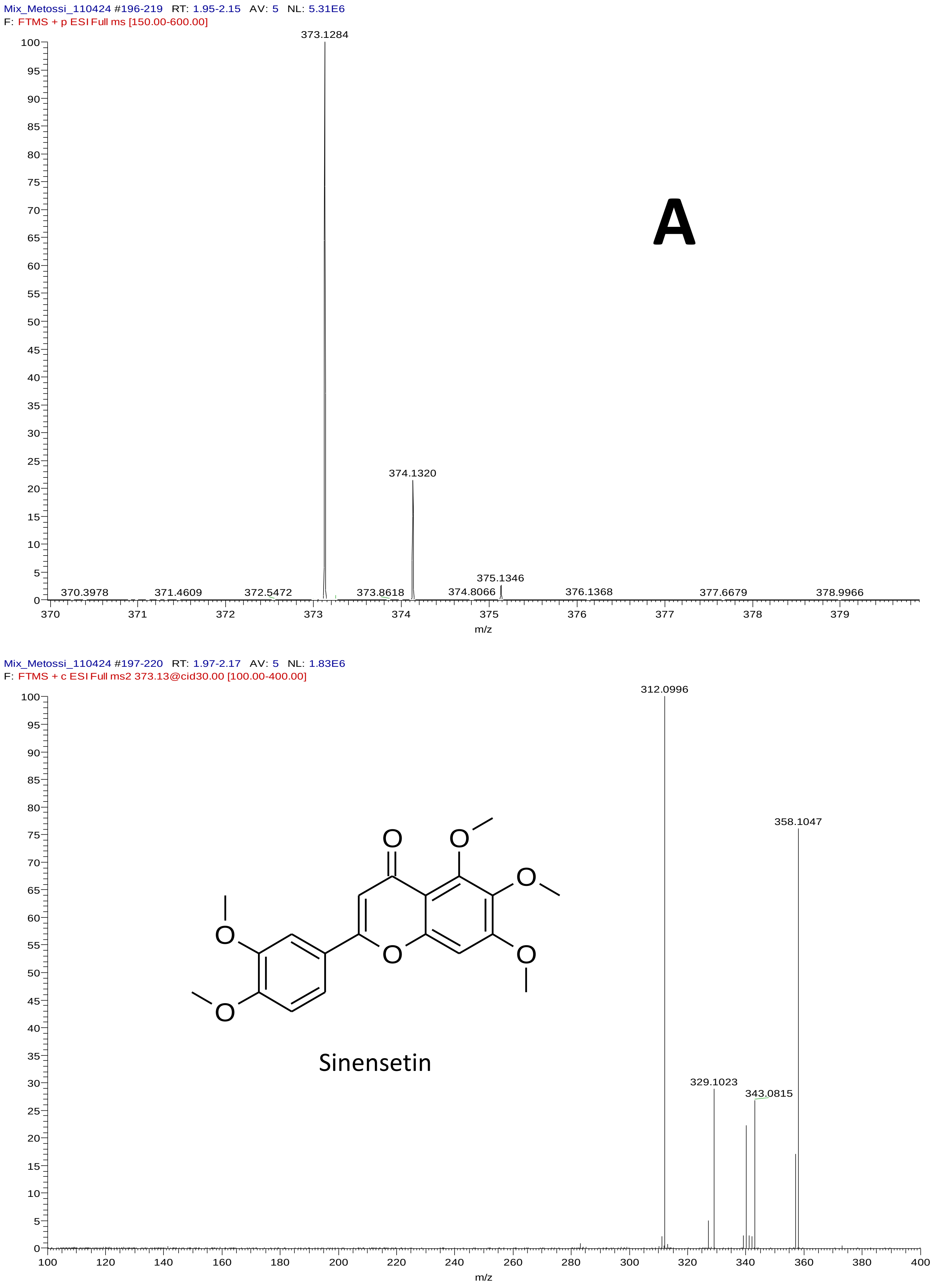

Mix_Metossi_110424 #1083-1110 RT: 10.90-11.10 AV: 5 NL: 5.03E
F: FTMS + p ESI Full ms [150.00-600.00]

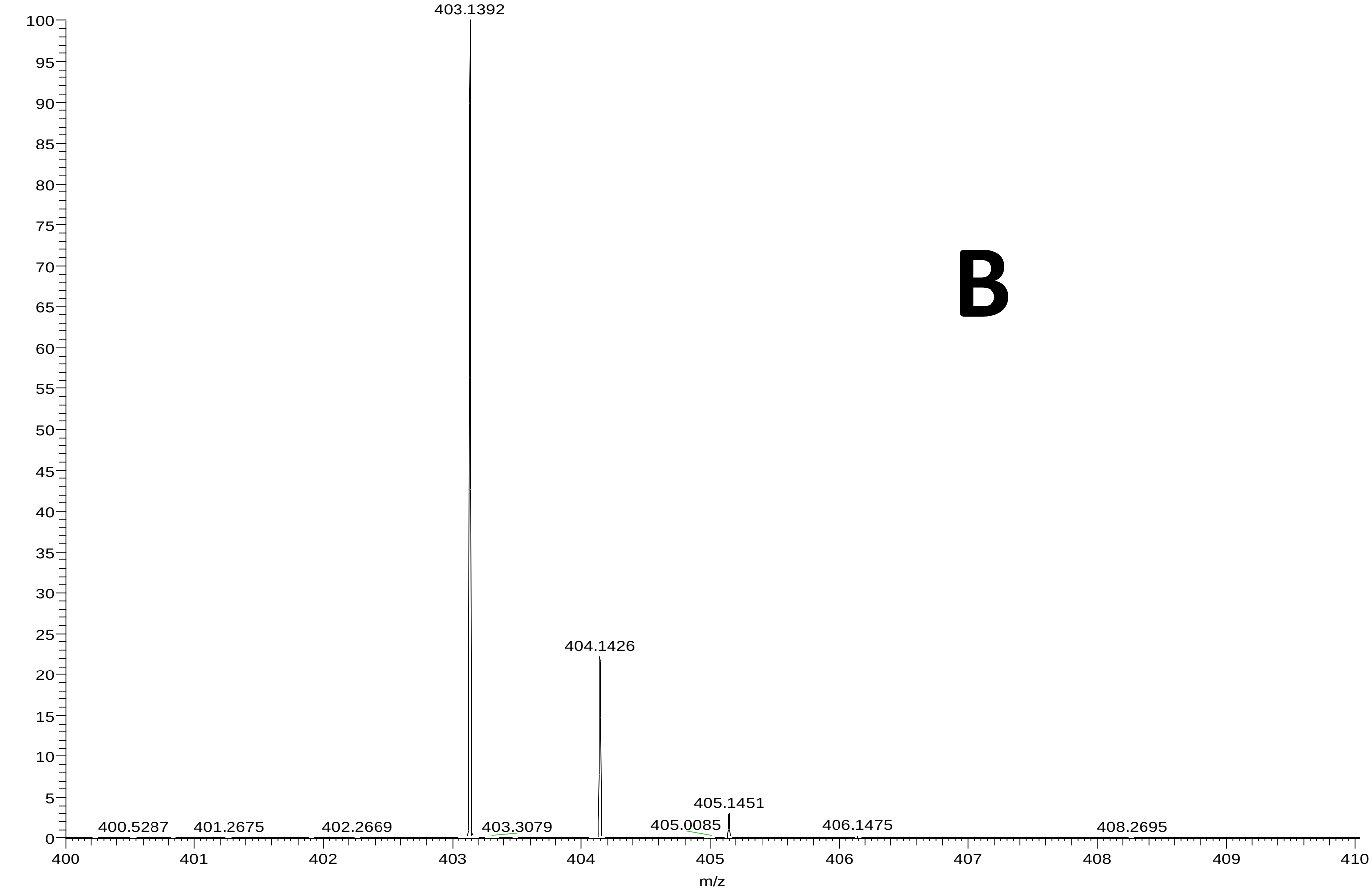

Mix_Metossi_110424 #173-197 RT: 1.73-1.93 AV: 5 NL: 6.78E4
F: FTMS + c ESI Full ms2 403.14@cid30.00 [110.00-420.00]

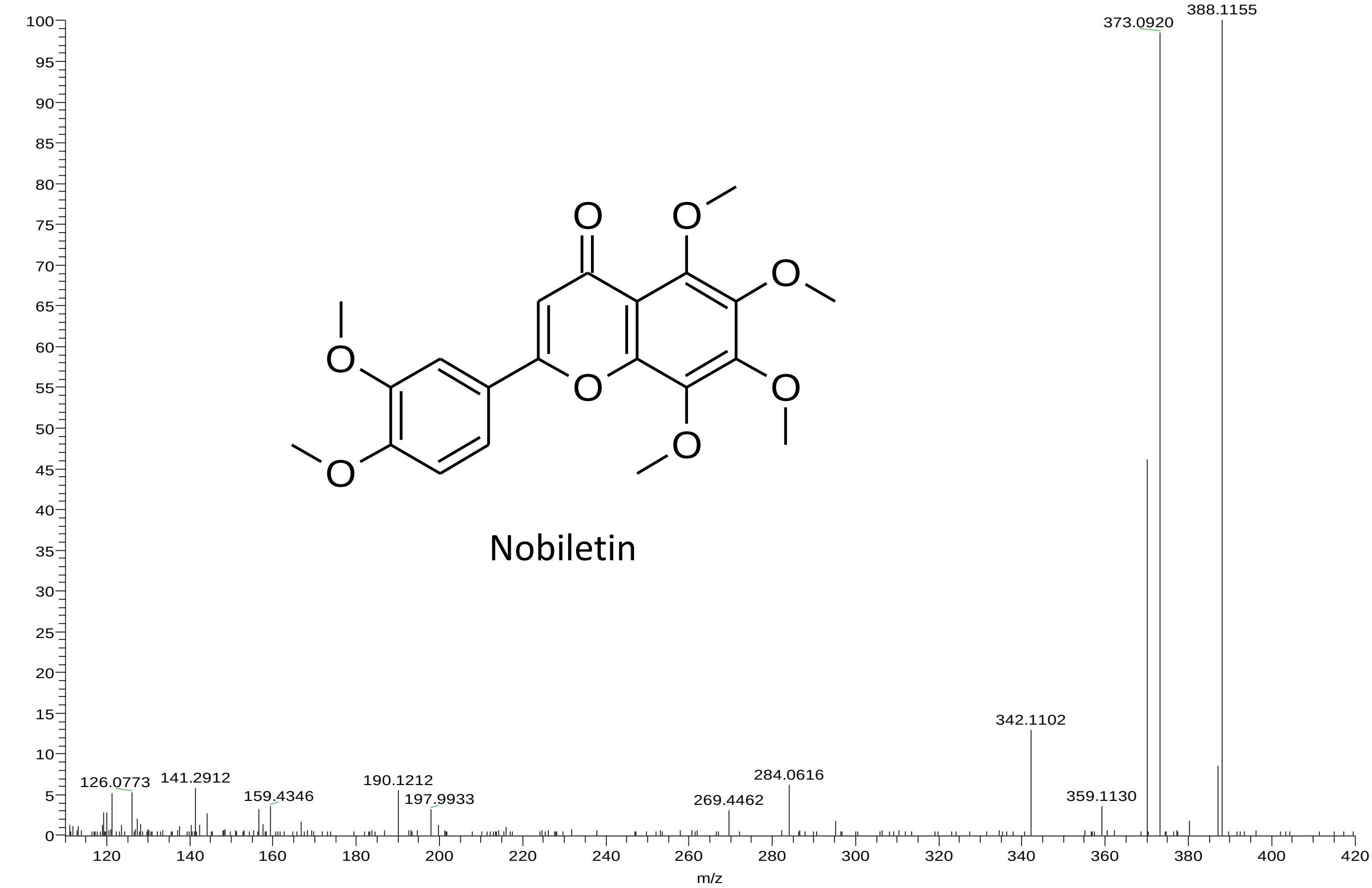

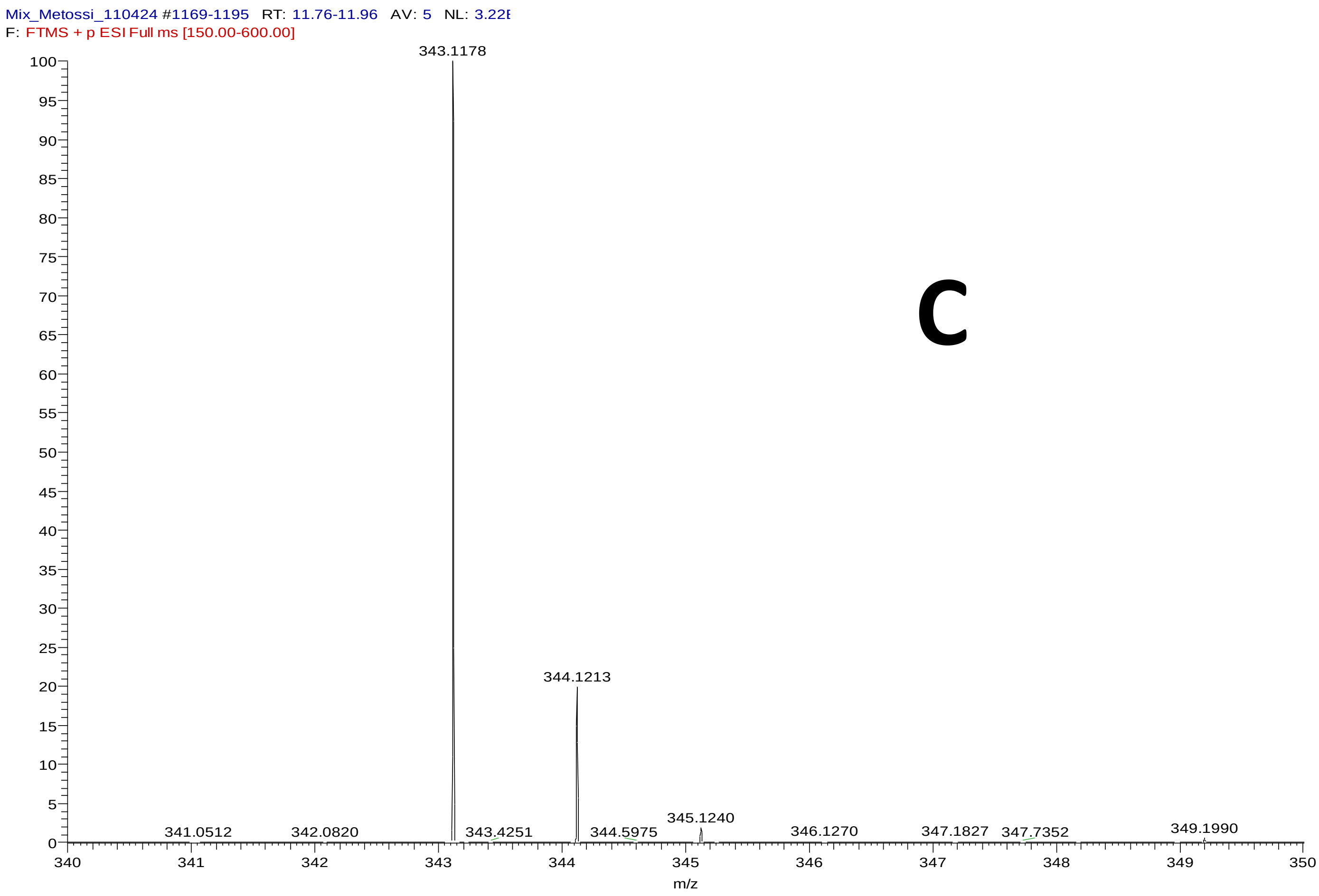
Mix_Metossi_110424 #1169-1195 RT: 11.76-11.96 AV: 5 NL: 3.22E
F: FTMS + p ESI Full ms [150.00-600.00]
343.1178
344.1213
345.1240
341.0512
342.0820
343.4251
344.5975
346.1270
347.1827
347.7352
349.1990
m/z
C

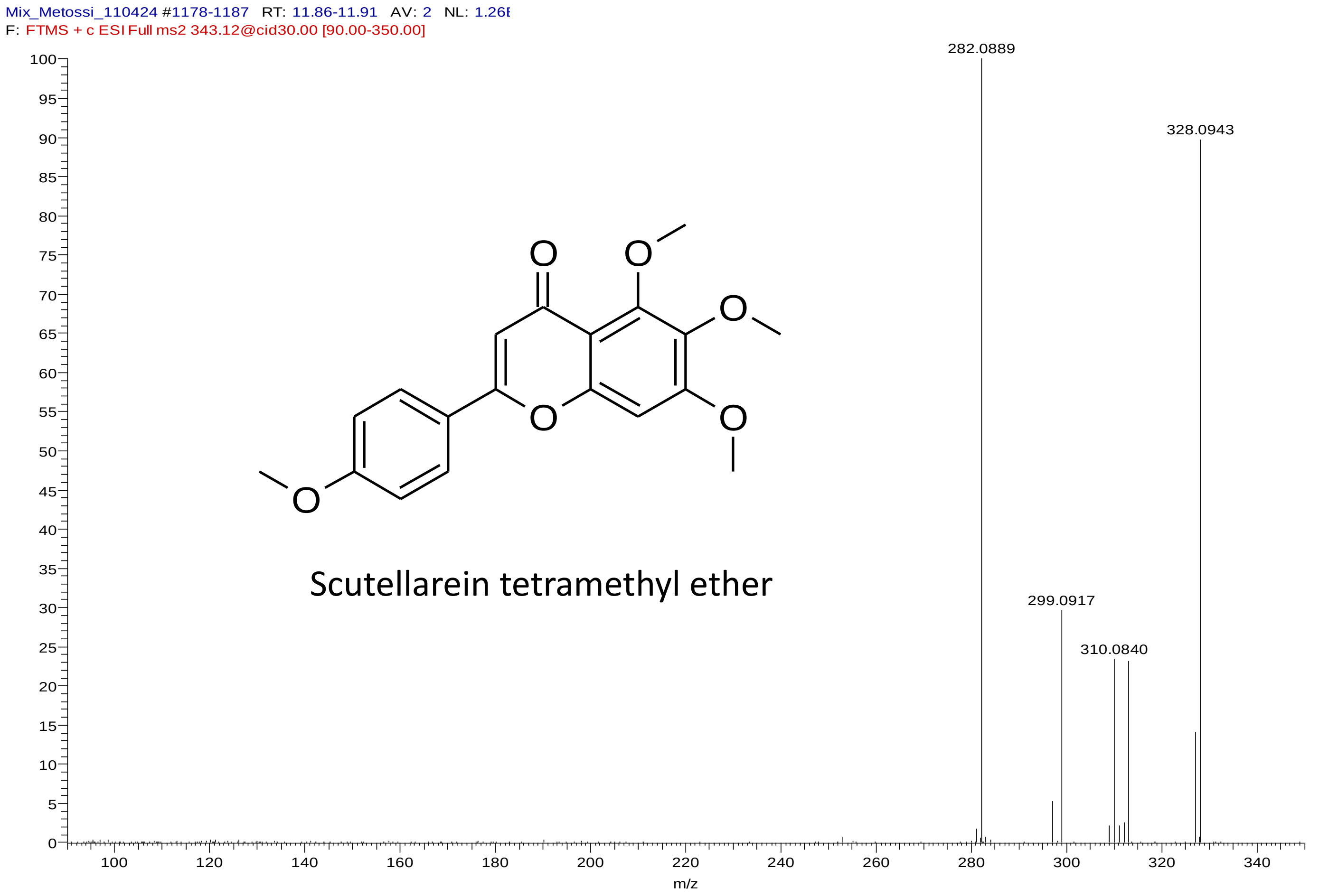
Mix_Metossi_110424 #1178-1187 RT: 11.86-11.91 AV: 2 NL: 1.26E
F: FTMS + c ESI Full ms2 343.12@cid30.00 [90.00-350.00]
282.0889
328.0943
299.0917
310.0840
m/z
Scutellarein tetramethyl ether

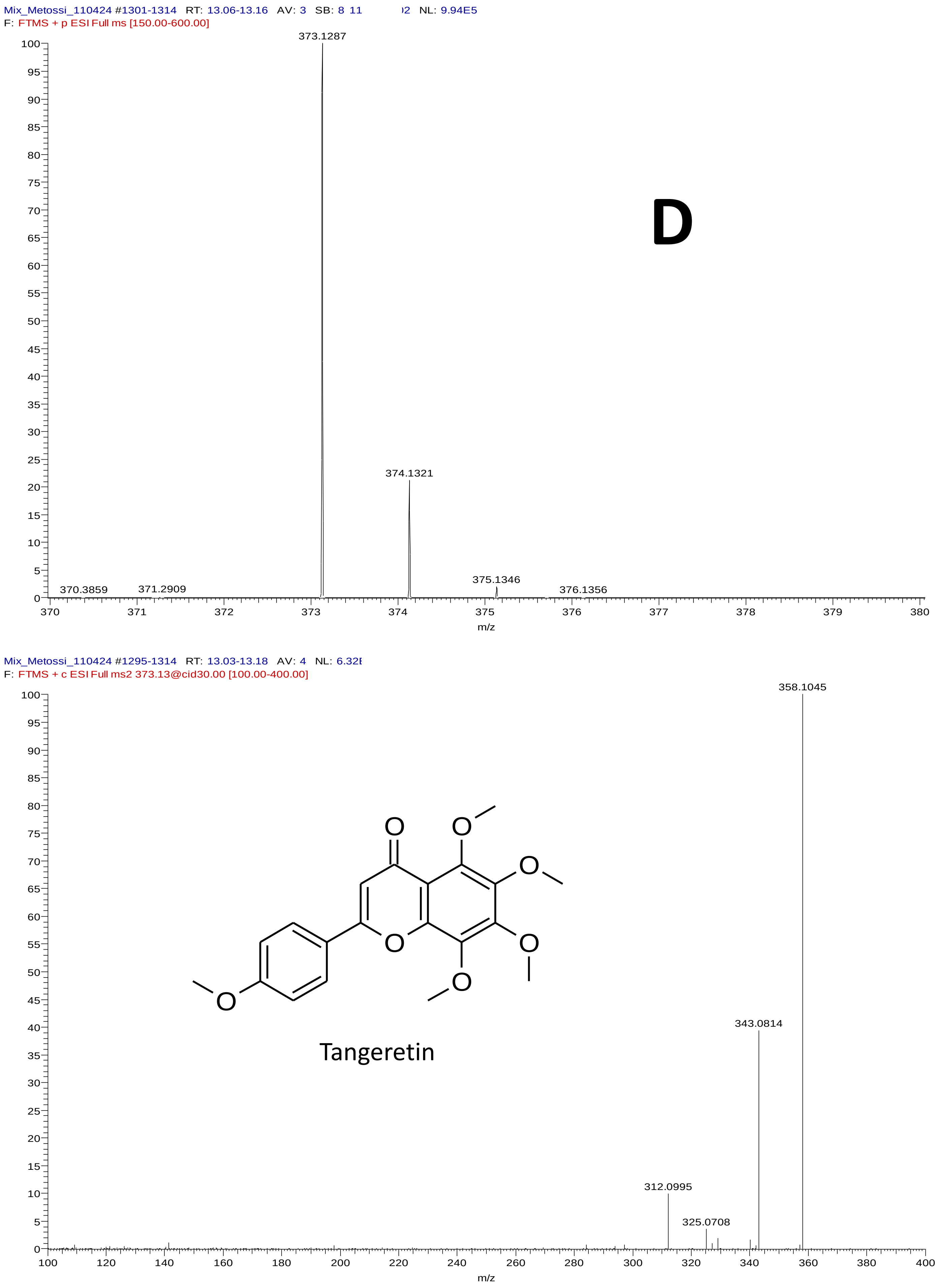
Mix_Metossi_110424 #1301-1314 RT: 13.06-13.16 AV: 3 SB: 8 11
NL: 9.94E5
F: FTMS + p ESI Full ms [150.00-600.00]
373.1287
374.1321
375.1346
376.1356
370.3859
371.2909
D
m/z
Mix_Metossi_110424 #1295-1314 RT: 13.03-13.18 AV: 4 NL: 6.32
F: FTMS + c ESI Full ms2 373.13@cid30.00 [100.00-400.00]
358.1045
343.0814
312.0995
325.0708
Tangeretin
m/z

Table **S1.** HRMS (ESI) *m/z* $[M+H]^+$ calculated and found for compounds reported in Figure S6.

Compound	Molecular formula	calcd $[M+H]^+$	Found	MMA (mDa)
sinensetin	$C_{20}H_{20}O_7$	373.1282	373.1284	0.23057
nobiletin	$C_{21}H_{22}O_8$	403.1387	403.1392	0.49591
tangeretin	$C_{20}H_{20}O_7$	373.1282	343.1178	0.23523
scutellarein TME	$C_{19}H_{18}O_6$	343.1176	373.1287	0.50057

Figure S7: in source ESI-CID spectra of four major component of the **M3** mixture compared with separated analytical standards. **A**) sinensetin, **B**) nobiletin, **C**) scutellarein tetramethyl ether, **D**) tangeretin.

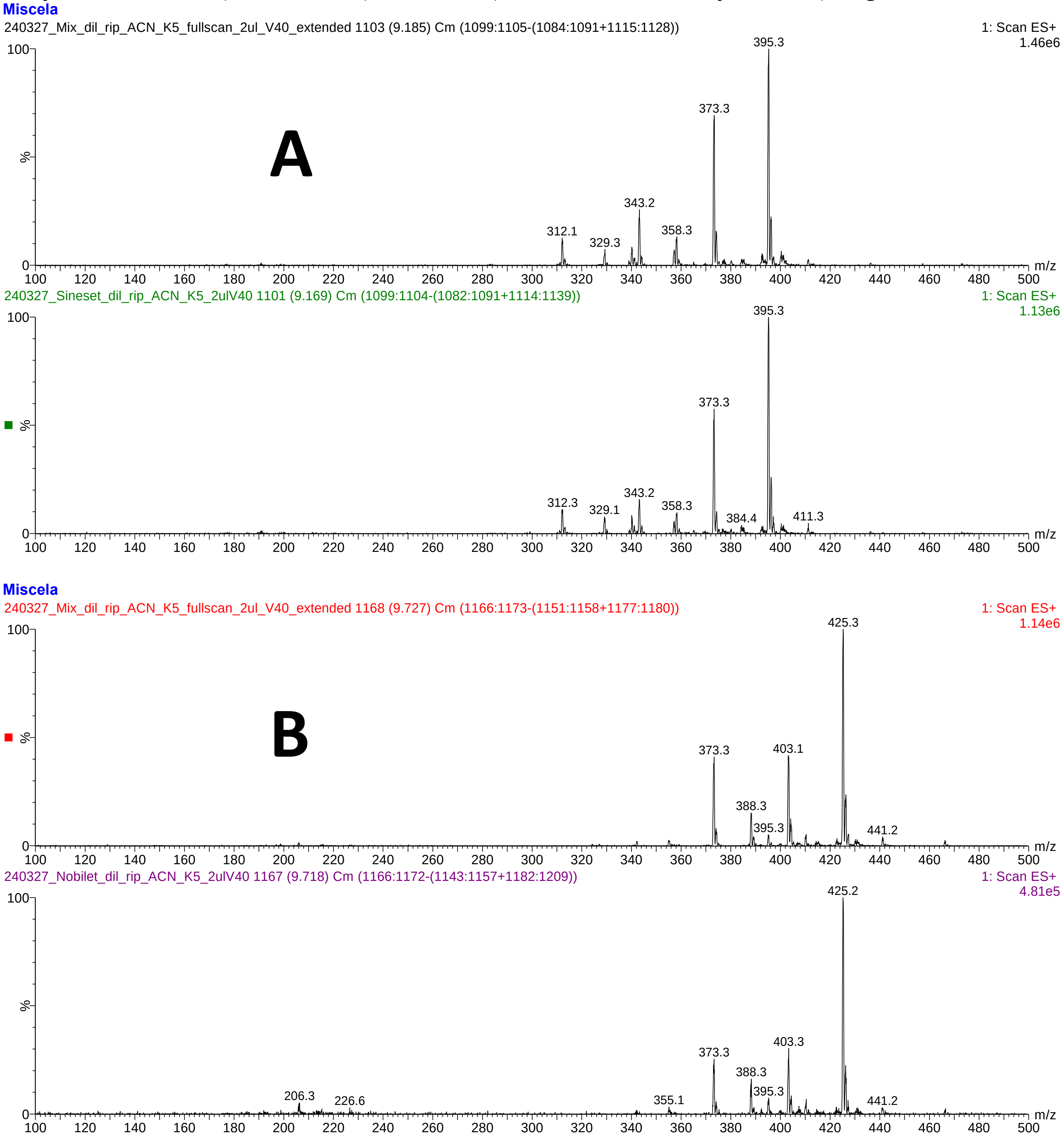

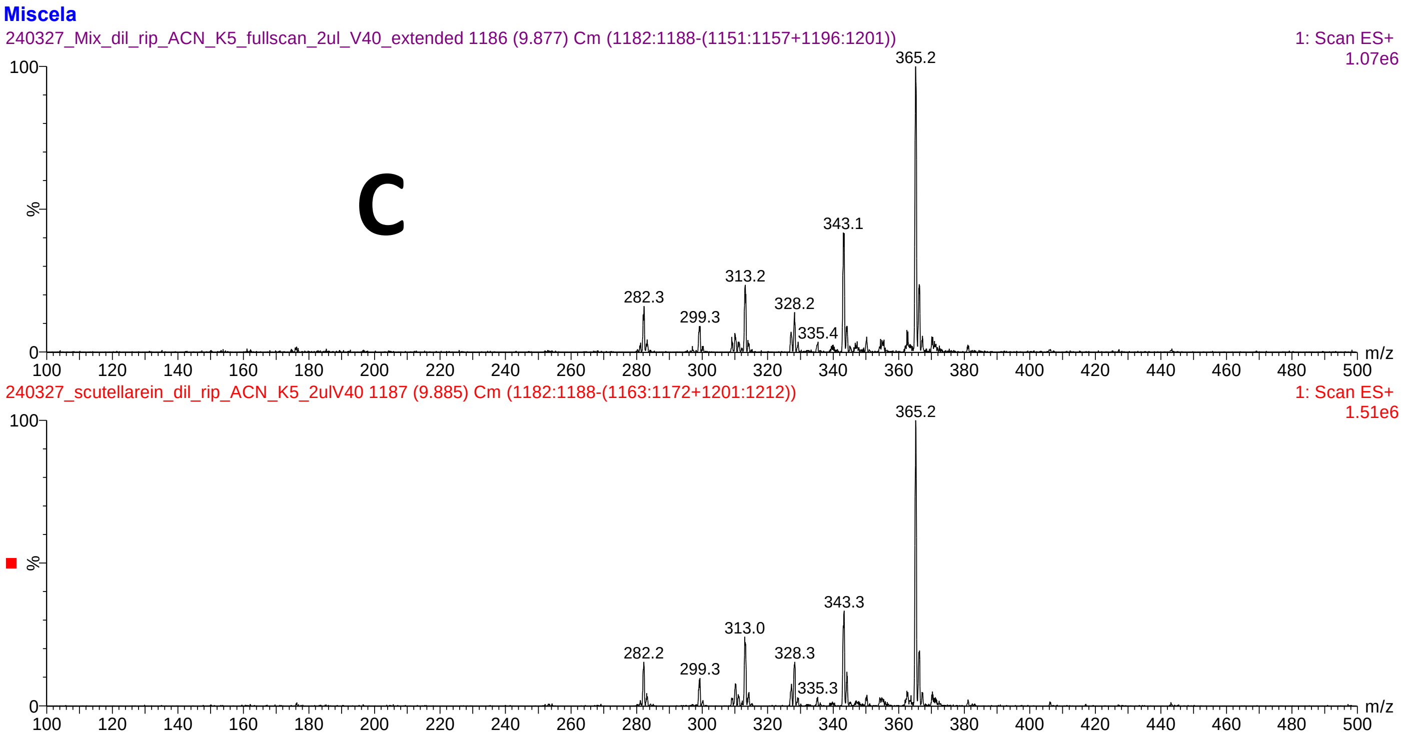
Miscela
240327_Mix_dil_rip_ACN_K5_fullscan_2ul_V40_extended 1186 (9.877) Cm (1182:1188-(1151:1157+1196:1201))
1: Scan ES+
1.07e6
C
365.2
343.1
313.2
282.3
299.3
328.2
335.4
240327_scutellarein_dil_rip_ACN_K5_2ulV40 1187 (9.885) Cm (1182:1188-(1163:1172+1201:1212))
1: Scan ES+
1.51e6
365.2
343.3
313.0
282.2
299.3
328.3
335.3
%
m/z

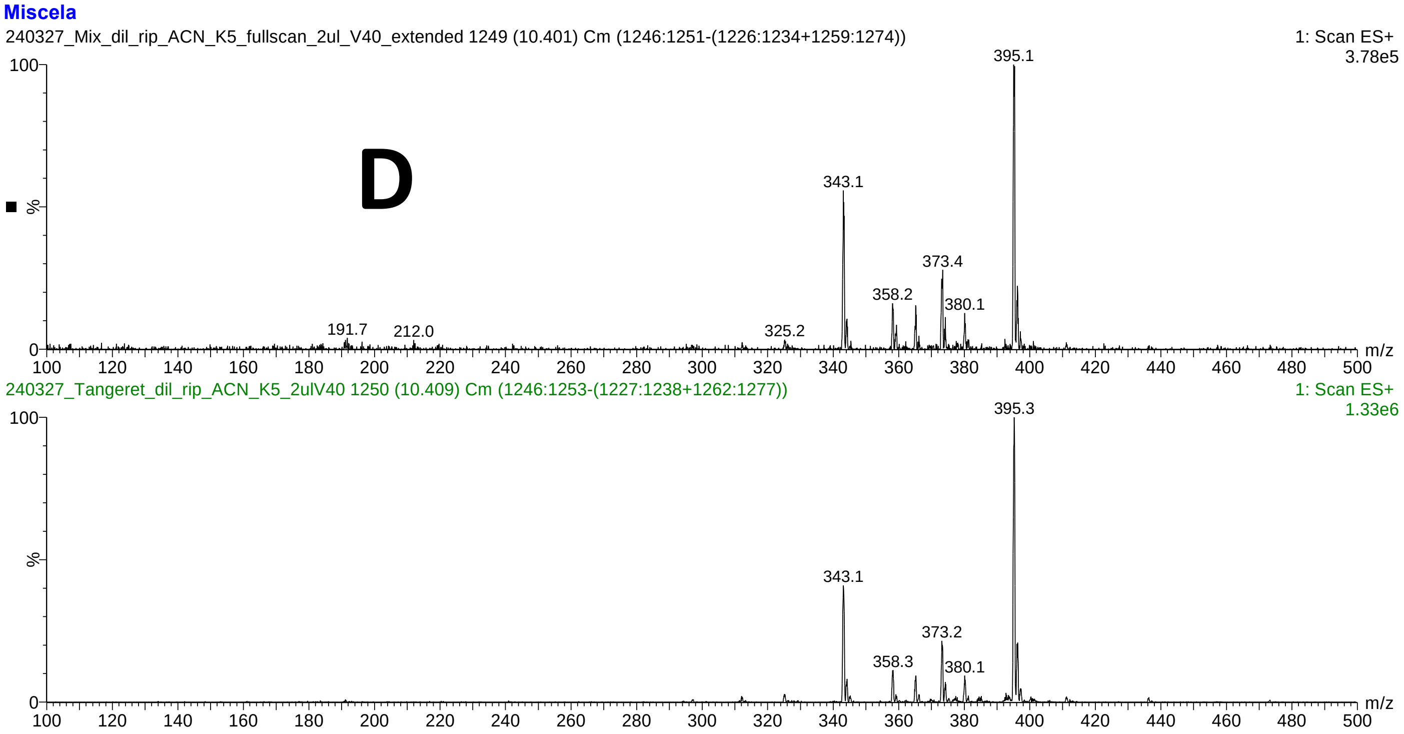
Miscela
240327_Mix_dil_rip_ACN_K5_fullscan_2ul_V40_extended 1249 (10.401) Cm (1246:1251-(1226:1234+1259:1274))
1: Scan ES+
3.78e5
D
395.1
343.1
373.4
358.2
380.1
191.7
212.0
325.2
240327_Tangeret_dil_rip_ACN_K5_2ulV40 1250 (10.409) Cm (1246:1253-(1227:1238+1262:1277))
1: Scan ES+
1.33e6
395.3
343.1
373.2
358.3
380.1
%
m/z

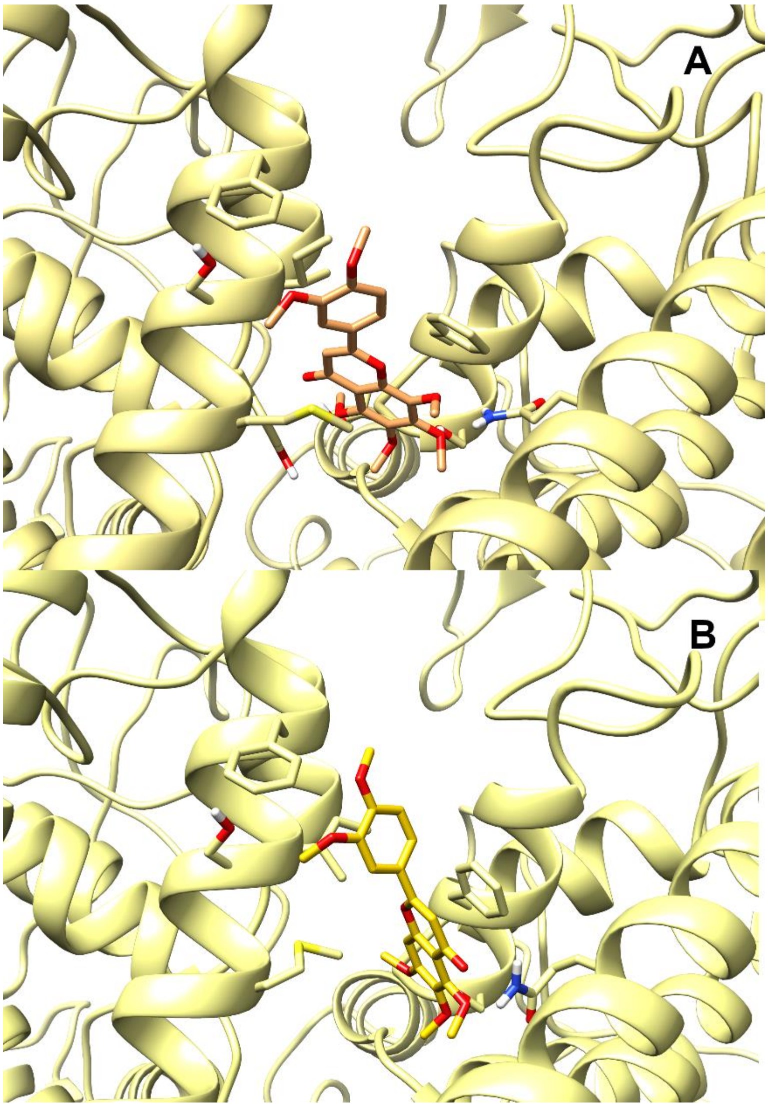

Figure S8. Representative docking poses of nobiletin (coloured in orange, panel A) and sinensetin (coloured in gold, panel B). Protein backbone atoms are represented as ribbons coloured in yellow. according to the side chains. Hydrogen, nitrogen, oxygen, and sulfur atoms are painted white, blue, red, and yellow, respec-tively
